# A standardized database of Chinese emotional short videos based on age and gender differences

**DOI:** 10.1371/journal.pone.0283573

**Published:** 2023-03-30

**Authors:** Danting Duan, Wei Zhong, Shuang Ran, Long Ye, Qin Zhang

**Affiliations:** 1 Key Laboratory of Media Audio & Video, Communication University of China, Beijing, China; 2 State Key Laboratory of Media Convergence and Communication, Communication University of China, Beijing, China; Nanyang Technological University, SINGAPORE

## Abstract

Most of the existing emotion elicitation databases use the film clips as stimuli and do not take into account the age and gender differences of participants. Considering the short videos have the advantages of short in time, easy to understand and strong emotional appeal, we choose them to construct a standardized database of Chinese emotional short videos by the joint analysis of age and gender differences. Two experiments are performed to establish and validate our database. In the Experiment 1, we selected 240 stimuli from 2700 short videos and analyzed the subjective evaluation results of 360 participants with different ages and genders. As a result, a total of 54 short videos with three categories of emotions were picked out for 6 groups of participants, including the male and female respectively aged in 20-24, 25-29 and 30-34. In the Experiment 2, we recorded the EEG signals and subjective experience scores of 81 participants while watching different video stimuli. Both the results of EEG emotion recognition and subjective evaluation indicate that our database of 54 short videos can achieve better emotion elicitation effects compared with film clips. Furthermore, the targeted delivery of specific short videos has also been verified to be effective, helping the researchers choose appropriate emotional elicitation stimuli for different participants and promoting the study of individual differences in emotion responses.

## Introduction

Emotion has always been a hot research topic in the fields of psychology and artificial intelligence [1–3]. As an essential step in affective computing, emotion elicitation has also drawn increasing attention [4]. Therefore, creating the effective emotion elicitation databases is becoming a popular topic for researchers interested in emotion.

Currently, the researchers have attempted a range of methods of eliciting emotion in the laboratory, such as interactive training, hypnosis, pictures, music, slides and film clips [5–7]. Compared to other emotional stimuli, the film clips (i.e., a portion of a full-length film) exhibit several advantages in emotion eliciting tasks. Firstly, the film clips are dynamic stimuli with both auditory and visual channels, which can highly attract attention [8]. Secondly, they have relatively high ecological validity and can induce strong subjective experiences and physiological changes [5]. Thirdly, the film clips present continuous emotional scenes and are able to capture the emotions that develop over time [9]. Furthermore, the meta-analysis of emotion elicitation has also validated that film clips are one of the most effective ways to elicit emotion [10]. Generally, the selection of film clips for emotion eliciting tasks should follow three criteria [5]: relatively short duration, comprehensible without additional explanation, and inducing a specific target emotion.

For the construction of emotional film databases, a lot of efforts have been made over the years. According to the emotion model [11], the existing emotional film databases can be divided into two categories, the dimensional model-based and the discrete model-based. The dimensional model believes that emotions are characterized by combinations of dimensions, such as valence, arousal, and dominance [12]. Baveye et al. [13] adopted a crowdsourcing approach by asking annotators to rate the degree of valence and arousal, and built a large free-shared database of 9,800 film clips. Zheng et al. [14] edited 15 film clips from 6 films, and divided their emotions into three categories (positive, neutral and negative) along the valence dimension. Ismail et al. [15] displayed 24 videos to 42 participants by an online survey, and the rating results of valence-arousal dimensions indicated that 79 percent of the videos could successfully elicit the target emotion. While the discrete model suggests that emotions can be classified into basic categories, and the first discrete emotional film database was developed in [16] to elicit six basic emotions proposed by Ekman [17]. Thereafter, Gross and Levenson [5] creatively proposed the concept of success index to quantify the effect of film clips on eliciting emotions and constructed a film database that can induce eight emotions. To provide more immersive experience, Jeong et al. [18] built a database of 4D films with the aid of chair movements, vibrations, winds and scents. It is worth noting that the participants in different cultures and languages will have different responses to the same emotional stimulus [19–21]. Therefore, the researchers have also created different databases for different cultures and languages. Michelini et al. [22] designed a film database for Latin-Americans, where the emotional states can be analyzed from both dimensional and discrete perspectives. Shalchizadeh et al. [23] recorded the emotional responses of 88 participants by means of the online website, and established a database of 21 film clips for Persian culture. Another film database was established for Asians in [24] that can induce eight emotions with each emotion type including 8 film clips. According to the self-assessment results of 50 college students on 30 Chinese film clips, 18 film clips were selected to form a standardized emotional film database in [25]. To further explore the structure of positive emotions, Zhang et al. [26] designed a database of 22 film clips containing four positive emotions (empathy, fun, creativity and esteem).

As a matter of fact, the individual difference of participants is an unavoidable influencing factor in the study of emotion elicitation. The emotional responses of different participants may vary greatly in the same elicitation situation. Numerous studies have shown that there are age-related differences in emotional responses [27–29]. Mather et al. [27] identified that for older adults (70–90 years), seeing positive pictures lead to stronger amygdala activation than negative ones, whereas this phenomenon was not found in younger adults (18–29 years). Buriss et al. [28] found that the older (66–95 years) and middle-aged (36–65 years) adults have higher valence in response to pleasant slides and lower valence to unpleasant ones compared to younger adults (18–35 years). Jenkins et al. [29] developed a database of verbal and non-verbal contemporary films for a large age range of participants, and concluded that regardless of watching positive or negative film clips, the participants aged 46–88 have stronger emotional responses than those aged 18–45. Similarly to age difference, there has also been a great deal of exploration of gender difference in emotional responses [30–32]. Bradley et al. [30] measured the emotional reactivity of males and females when viewing emotional pictures, and suggested that the female shows greater defensive responses to aversive pictures, whereas the male shows stronger appetite activation only when viewing pornography. The difference in sensitivity to negative stimuli was studied between males and females in [31], and the experimental results showed that the female has greater sensitivity to negative stimuli. Deng et al. [32] explored gender differences in terms of both emotional experience and emotional expression, and concluded that the male typically has stronger emotional experiences, while the female is better at expressing emotions, especially negative emotions. From the above analysis, it can be concluded that although the researchers have designed various experiments exploring the age and gender differences in emotional responses, the issue of individual differences has not been considered in the construction of emotion elicitation databases. Therefore, it is necessary to pay attention to the age, gender and cultural background of participants and provide specific emotion-evoking stimuli for different participants.

On the other hand, how to evaluate the quality of an emotion elicitation database? The most common method is to ask participants to rate the elicited material according to their feelings during the emotion elicitation phase. Unfortunately, it is difficult to describe emotions accurately and quantitatively by relying only on subjective assessment. It has been shown that the occurrence of emotions is usually accompanied by changes in physiological signals, such as galvanic skin response (GSR), heart rate (HR), electrocardiogram (ECG) and electroencephalography (EEG) signals [33]. Compared with other physiological signals, EEG can directly record the changes of scalp potentials and reflect the emotions more objectively and accurately, and thus it has been widely used in the field of affective computing [34–36]. Koelstra et al. [37] used 32 active AgCl electrodes to capture the EEG signals of participants while watching music videos, and a significant correlation was found between participant ratings and EEG frequencies. A high-density EEG signal database was built by using a 128-channel EEG device in [38], and a feature selection method was also presented for emotion recognition. In recent years, several consumer-grade EEG devices have also been developed with the advantages of low cost, good portability and reliability, such as Emotiv, OpenBCI and NeuroSky [39]. Liu et al. [40] used the Emotiv EPOC with 14 electrodes to record the EEG signals of participants while watching film clips. Stylianos et al. [41] developed an OpenBCI-based software for detecting, displaying and analyzing EEG signals. Therefore, considering EEG signals can reflect the emotions more authentically, the effect of emotion elicitation by stimuli database can be evaluated through the accuracy of EEG emotion recognition.

Taking into account the individual difference, the main purpose of this paper is to build a standardized Chinese emotion elicitation database for participants of different ages and genders. Considering the advantages of short duration, easy understanding and strong emotional appeal, we choose the short videos as emotion-evoking stimuli. For constructing the database of Chinese emotional short videos, we develop three hypotheses: 1) the individual differences in age and gender will lead to differences in emotional responses when watching emotional short videos; 2) compared to film clips, the stimuli of short videos will bring better emotion elicitation effect; 3) the specific short videos designed for participants of different ages and genders contribute to better emotion elicitation effect than those do not account for individual differences. Compared with the existing emotion elicitation databases, our database has the following characteristics: 1) it is the first attempt to use short videos as emotion-evoking materials; 2) the age and gender differences of participants are considered jointly for the first time to provide specific short videos for different groups; 3) the subjective and physiological responses of participants are recorded simultaneously, and the EEG signals are used for emotion recognition to evaluate the quality of our emotion elicitation database.

## Experiment 1

The purpose of Experiment 1 is to explore whether there are age and gender differences in emotional responses when watching short videos, and pick out the short videos for constructing our emotion elicitation database that are more likely to elicit emotion for different groups.

### Methods

#### Ethics

The experimental procedures were approved by the Institutional Review Board of State Key Laboratory of Media Convergence and Communication of Communication University of China (CUCE-2022–017). All participants voluntarily agreed to participate in this study and signed the written informed consent to have data from their records used in research and publication of these case details. All data have been fully anonymized before we accessed them, and the database can be used only for academic research upon request for approval.

#### Materials

In recent years, with the rapid development of mobile Internet technology, the short video industry has been rising rapidly. In fact, a large number of short video databases have been proposed and used for different tasks, such as target detection [42], behavior recognition [43] and person identification [44]. However, to the best of our knowledge, the short videos have not been used for the construction of emotion elicitation databases so far. We believe that the short videos may be more suitable for emotion elicitation compared to film clips. Firstly, as the name suggests, the short videos are short in length and can be directly used as emotion elicitation materials without secondary editing. Secondly, the short videos are carefully edited, compact in plot and easy to understand. Thirdly, the short videos have specific themes and can bring strong emotional impact in a short time.

The short videos used in this experiment were obtained as follows. The 30 research assistants aged in 20–24, 25–29 and 30–34 were trained in the definition and subjective assessment of different emotions, with each age group including 5 males and 5 females. They were asked to select short videos that might elicit eight categories of emotions according to [45], i.e., four negative emotions (disgust, anger, fear and sadness), neutrality and three positive emotions (tenderness, amusement and joy). We specified the following criteria for the selection of short videos: 1) 60–240 seconds in duration; 2) easy to understand without additional explanation; 3) eliciting a specific emotion; 4) Chinese cultural and language; and 5) horizontal screen. After the subjective selection by assistants, the 2,700 short videos (duration: 60–236 seconds, mean = 148.69 seconds) were selected and labeled with the emotional categories. audio tracks. Then, these short videos were viewed independently by four cognitive psychologists aged in 32–45 (2 males and 2 females) with experience in affective assessment. For each short video, the cognitive psychologists subjectively assessed its potential to successfully elicit one and only one of the target emotion categories. Only the short videos that were unanimously satisfactory to all four cognitive psychologists were retained, thus giving 240 short videos (duration: 71–232 seconds, mean = 150.06 seconds) for further study. It is worth noting that the emotional categories of short videos would not be changed after the evaluation of the four cognitive psychologists. The number of short videos for each emotion category ranged from 25 to 35.

#### Participants

We recruited the volunteers on the campus of Communication University of China by releasing a poster. Considering the participants who are extroverted and emotionally stable are more likely to be induced with the target emotion, we used the Eysenck Personality Questionnaire Short Scale for Chinese (EPQ-RSC) [46] to choose the volunteers. The EPQ-RSC conceptualizes personality into four dimensions: Psychoticism, Extraversion, Neuroticism and Lie. Different tendencies and degrees of expression in these four dimensions constitute different personality traits. A total of 482 volunteers enrolled in this recruitment, and we finally selected 360 participants based on EPQ-RSC. These participants are aged in three groups of 20–24, 25–29 and 30–34, with each age group including 60 males and 60 females. All of them are healthy right-handed, and not majored in psychology. Prior to the formal experiment, all participants were informed of the experimental procedure. Each participant received 120 RMB as compensation.

#### Measures

*1) Self-assessment 9-point scale*. In the subjective evaluation stage, we designed a Self-assessment 9-point scale to evaluate the degrees of valence and arousal of participants when watching short videos. This scale is adapted from the Self-assessment manikin (SAM) [47] that measures the degree of emotional responses in the form of pictures. The scale for this experiment considers the ratings of both valence (from very negative to very positive) and arousal (from very calm to very excited) associated with each short video. Each aspect was assessed on a 9-point Likert scale, meaning that the participants were asked to choose the score from 1 to 9 that best matched their true feelings when watching the short videos. Furthermore in order to avoid understanding bias and obtain more uniform results, we gave each participant an additional guide for their reference which clearly defined the meaning of each score.

*2) Emotional evaluation scale.* To investigate the intensity of each emotional dimension, we developed an emotional evaluation scale adapted from the Differential Emotions Scale (DES) [16] that measures the differentiation component of emotions. The scale includes three emotional states, i.e., positive, neutral and negative. The participants were asked to score the emotional intensity of each state on a 9-point Likert scale (from not at all to extremely) according to their real feelings when watching short videos. The ratings of this scale were used later to calculate the success index, which is an objective criterion for selecting the effective emotional stimuli.

#### Procedure

The experiment was conducted in an online format over a period of 6 days. The selected 240 short videos were pseudo-randomly assigned to six subsets and the number of short videos with the same emotion category was at most six in each subset. Correspondingly, the 360 participants were also divided into six groups, and the participants of each group are aged respectively in 20–24, 25–29 and 30–34 with each age set including 10 males and 10 females. For each day, one of six groups of participants attended an online meeting at a fixed time (2:00–5:00 pm) in a bright and quiet environment. The participants were asked to watch one subset of short videos, which were randomized in order. In particular, no more than two short videos of the same emotion category or three short videos of the same valence state, were shown consecutively. In this experiment, we did not let the participants watch these short videos in advance, and the participants also indicated they have not watched these short videos within one month. Before the formal experiment, an experimenter introduced the experimental procedure and scoring criteria of above two scales. Firstly, a 30-second blank screen was presented before playing a video, helping the participants empty their brains of all thoughts, feelings and memories. Next, the participants were asked to watch each short video carefully, they could move their eyes away from the screen if the video content makes them feel strongly uncomfortable. After watching each short video, the participants were encouraged to complete the above two scales based on their immediate true feelings. At the end of the experiment, the experimenter explained that the purpose was to examine the differences in emotional responses among participants of different ages and genders when watching short videos. Finally, each participant watched 40 short videos and each short video was viewed by 60 participants.

#### Data analysis

The results of each participant were averaged across the short videos for each emotion category. The scores of valence and arousal were examined in two separate three-way mixed analyses of variance (ANOVAs), including age (20–24, 25–29 and 30–34), gender (male and female) and emotion category (disgust, anger, fear, sadness, neutrality, tenderness, amusement and joy). The age and gender were between-subject factors, while the emotion category was a within-subject factor. The level of statistical significance was set at *p* < 0.05. And the effect sizes were presented as partial eta squared (*η*^2^) for ANOVA effects. It has been verified that the valence scores and arousal scores of each emotion category obey the normal distribution, and the multiple pairwise comparisons were implemented by using Bonferroni’s correction. All the analyses were performed by using IBM SPSS Statistics 25.0 software.

### Results

#### Valence effects

For each emotion category scored by participants of different ages and genders, the descriptive statistical results on the valence dimension are shown in Table 1. The mean score (*M*) and standard deviation (*SD*) were calculated to evaluate the valence degree of participants elicited by short videos.

**Table 1:** The descriptive statistical results on the valence dimension.

		Disgust	Anger	Fear	Sadness	Neutrality	Tenderness	Amusement	Joy	Total
20–24	Male	2.28 (0.81)	2.40 (0.44)	1.89 (0.59)	2.72 (0.61)	4.75 (0.70)	6.35 (0.90)	6.08 (0.74)	6.40 (0.95)	4.11 (2.00)
	Female	1.89 (0.65)	2.88 (0.82)	2.43 (0.82)	1.92 (0.65)	5.12 (0.60)	6.86 (0.93)	6.34 (0.90)	6.62 (0.98)	4.26 (2.21)
	Total	2.09 (0.76)	2.64 (0.70)	2.16 (0.76)	2.32 (0.74)	4.93 (0.68)	6.61 (0.95)	6.21 (0.83)	6.51 (0.96)	4.18 (2.11)
25–29	Male	2.85 (0.84)	2.57 (0.72)	2.80 (0.98)	2.03 (0.68)	4.69 (0.89)	6.22 (0.85)	6.56 (1.03)	6.29 (0.96)	4.25 (1.98)
	Female	1.99 (0.64)	2.80 (0.64)	2.61 (1.04)	2.34 (0.81)	4.94 (0.96)	6.24 (0.87)	6.81 (0.77)	6.58 (0.90)	4.29 (2.11)
	Total	2.42 (0.86)	2.68 (0.69)	2.71 (1.01)	2.19 (0.76)	4.81 (0.93)	6.23 (0.86)	6.68 (0.92)	6.44 (0.94)	4.27 (2.04)
30–34	Male	2.64 (0.75)	2.55 (0.93)	2.05 (0.66)	2.08 (0.77)	4.75 (1.04)	6.09 (0.62)	6.80 (0.93)	6.34 (0.61)	4.16 (2.08)
	Female	2.07 (0.79)	2.87 (0.86)	2.50 (0.87)	2.22 (0.78)	4.93 (0.88)	6.59 (0.71)	6.77 (1.04)	6.62 (1.00)	4.32 (2.17)
	Total	2.35 (0.82)	2.71 (0.90)	2.27(0.80)	2.15 (0.78)	4.84 (0.96)	6.34 (0.71)	6.78 (0.98)	6.48 (0.84)	4.24 (2.13)
Total	Male	2.59 (0.83)	2.51 (0.73)	2.24 (0.85)	2.27 (0.76)	4.73 (0.88)	6.22 (0.80)	6.48 (0.95)	6.35 (0.85)	4.17 (2.02)
	Female	1.98 (0.70)	2.85 (0.78)	2.51 (0.91)	2.16 (0.76)	4.99 (0.83)	6.56 (0.88)	6.64 (0.93)	6.60 (0.95)	4.29 (2.16)
	Total	2.29 (0.82)	2.68 (0.77)	2.38 (0.89)	2.22 (0.76)	4.86 (0.87)	6.39 (0.86)	6.56 (0.94)	6.47 (0.91)	4.23 (2.09)

**Fig 1:**
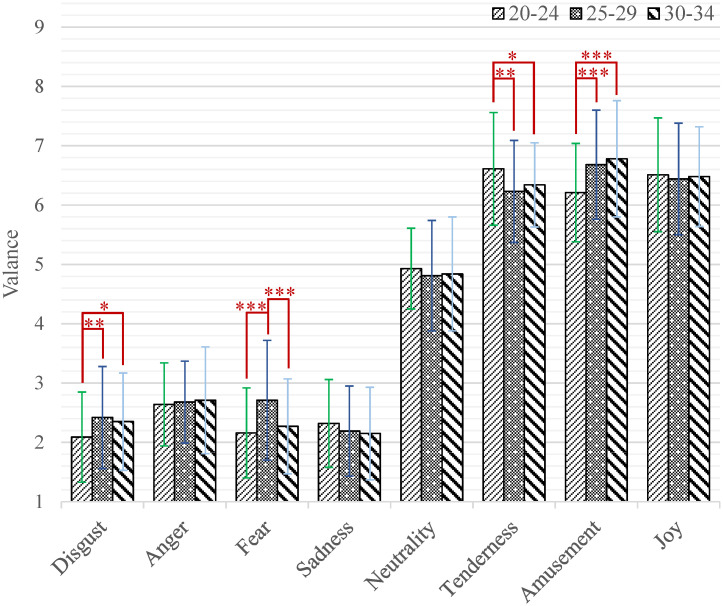
The valence scores of each category of emotion-inducing short videos among participants with different ages. Statistical significance: *p < 0.05, **p < 0.01, ***p < 0.001. Unless marked with the asterisk, no significant differences between these groups were found.

When processing the three-way mixed ANOVA on the valence dimension, there was a significant main effect of emotion category (*F*_(7,2478)_ = 2237.96, *p* < 0.001, *η*^2^ = 0.86). The *post-hoc* test indicated that there were no significant differences among the short videos with three positive emotions (tenderness, amusement and joy), but they were significantly different from those with other emotion categories. All the valence scores of positive short videos were significantly higher than those of neutral ones, and the valence scores of neutral videos were significantly higher than those of negative ones. More specifically, the valence scores of emotion categories differed significantly as follows: sadness, disgust, fear < anger (*p* < 0.001) < neutrality (*p* < 0.001) < tenderness, joy, amusement (*p* < 0.001). The interaction effect of emotion category by age (*F*_(14,2478)_ = 6.20, *p* < 0.001, *η*^2^ = 0.03) was significant. Further, the results of simple effect analysis are shown in Fig 1. The participants aged 20–24 gave significantly higher valence scores than those aged 25–29 while watching short videos that induced tenderness (*M* = 6.61, *SD* = 0.95 versus *M* = 6.23, *SD* = 0.86; *p* = 0.001). The participants aged 20–24 gave significantly higher valence scores than those aged 30–34 while watching short videos that induced tenderness (*M* = 6.61, *SD* = 0.95 versus *M* = 6.34, *SD* = 0.71; *p* = 0.035). The participants aged 25–29 gave significantly higher valence scores than those aged 20–24 while watching short videos that induced disgust (*M* = 2.42, *SD* = 0.86 versus *M* = 2.09, *SD* = 0.76; *p* = 0.002), fear (*M* = 2.71, *SD* = 1.01 versus *M* = 2.16, *SD* = 0.76; *p* < 0.001) and amusement (*M* = 6.68, SD = 0.92 versus *M* = 6.21, *SD* = 0.83; *p* < 0.001). The participants aged 25–29 gave significantly higher valence scores than those aged 30–34 while watching short videos that induced fear (*M* = 2.71, *SD* = 1.01 versus *M* = 2.27, *SD* = 0.80; *p* < 0.001). The participants aged 30–34 gave significantly higher valence scores than those aged 20–24 while watching short videos that induced disgust (*M* = 2.35, *SD* = 0.82 versus *M* = 2.09, *SD* = 0.76; *p* = 0.019) and amusement (*M* = 6.78, *SD* = 0.98 versus *M* = 6.21, *SD* = 0.83; *p* < 0.001).

**Fig 2:**
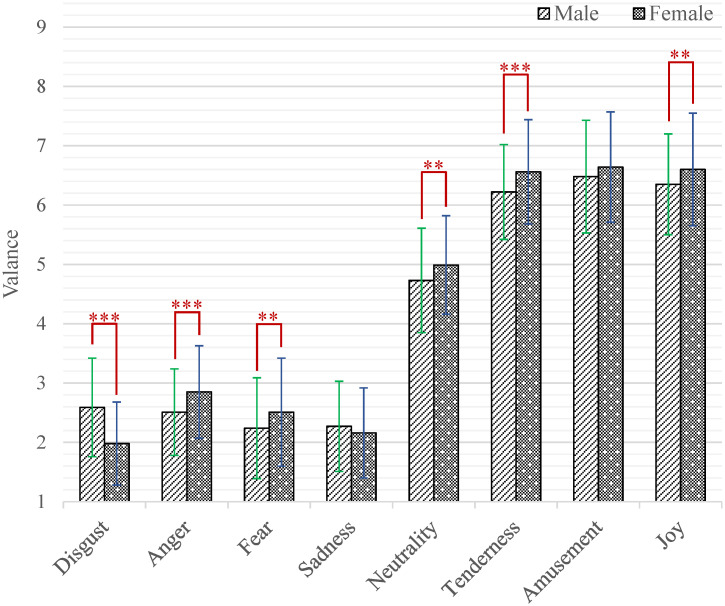
The valence scores of each category of emotion-inducing short videos between male and female. Statistical significance: *p < 0.05, **p < 0.01, ***p < 0.001. Unless marked with the asterisk, no significant differences between these groups were found.

At the same time, the interaction effect of emotion category by gender (*F*_(7,2478)_ = 14.28, *p* < 0.001, *η*^2^ = 0.04) was also significant. Further, the results of simple effect analysis are shown in Fig 2. The male had higher valence scores than female while watching short videos that induced disgust (*M* = 2.59, *SD* = 0.83 versus *M* = 1.98, *SD* = 0.70; *p* < 0.001). While the female had higher valence scores than male when inducing anger (*M* = 2.85, *SD* = 0.78 versus *M* = 2.51, *SD* = 0.73; *p* < 0.001), fear (*M* = 2.51, *SD* = 0.91 versus *M* = 2.24, *SD* = 0.85; *p* = 0.003), neutrality (*M* = 4.99, *SD* = 0.83 versus *M* = 4.73, *SD* = 0.88; *p* = 0.004), tenderness (*M* = 6.56, *SD* = 0.88 versus *M* = 6.22, *SD* = 0.80; *p* < 0.001) and joy (*M* = 6.60, *SD* = 0.95 versus *M* = 6.35, *SD* = 0.85; *p* = 0.007).

**Fig 3:**
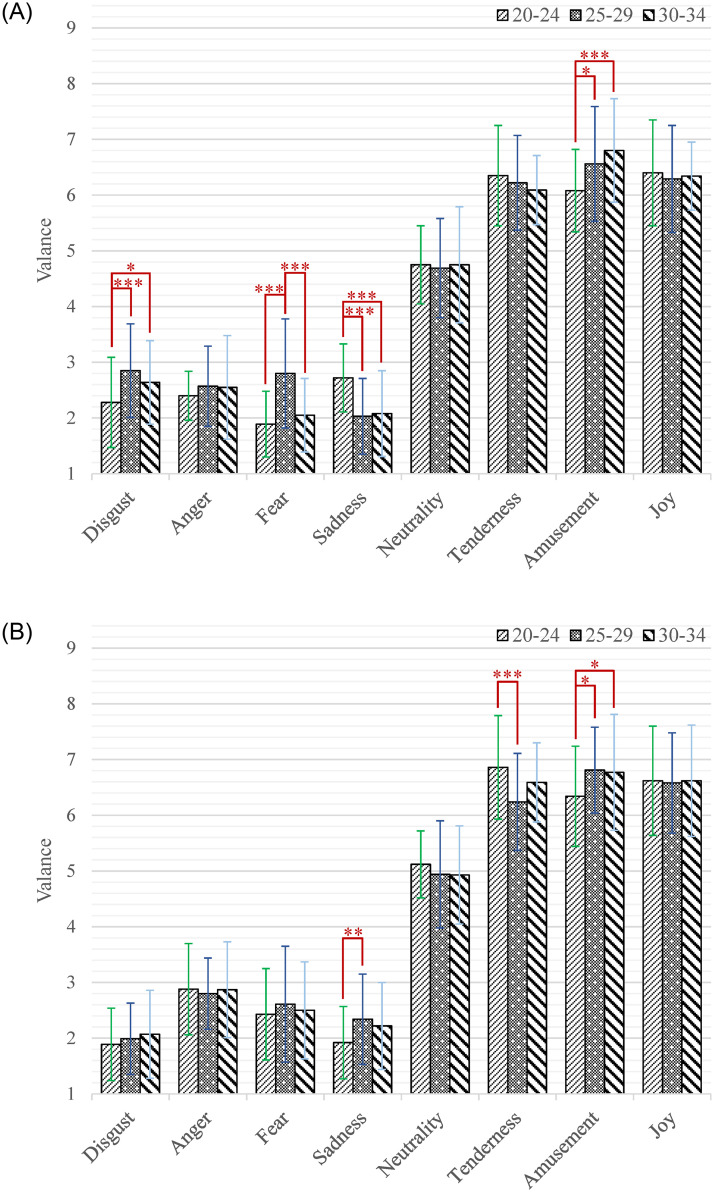
The valence scores of each category of emotion-inducing short videos among participants with different ages. A: Male. B: Female. Statistical significance: *p < 0.05, **p < 0.01, ***p < 0.001. Unless marked with the asterisk, no significant differences between these groups were found.

**Fig 4:**
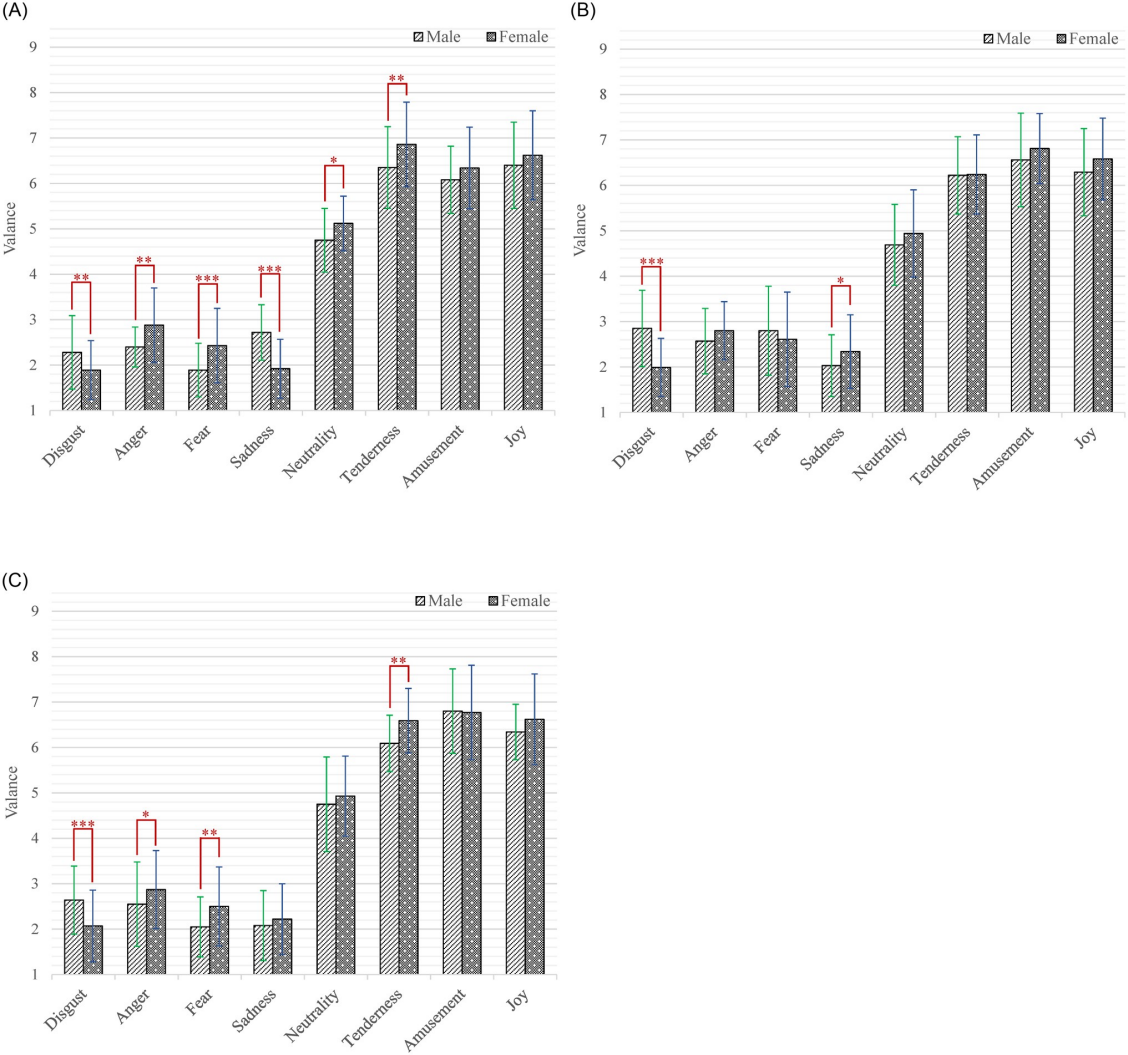
The valence scores of each category of emotion-inducing short videos between male and female. A: Age = 20–24. B: Age = 25–29. C: Age = 30–34. Statistical significance: *p < 0.05, **p < 0.01, ***p < 0.001. Unless marked with the asterisk, no significant differences between these groups were found.

Moreover, the interaction effect of emotion category by age by gender (*F*_(14,2478)_ = 4.26, *p* < 0.001, *η*^2^ = 0.02) was significant. According to our research interests, the simple-simple effect analysis of age on gender and emotion category was firstly conducted. As shown in Fig 3A, among the male participants, the participants aged 20–24 gave significantly higher valence scores than those aged 25–29 while watching short videos that induced sadness (*M* = 2.72, SD = 0.61 versus *M* = 2.03, SD = 0.68; *p* < 0.001). The participants aged 20–24 gave significantly higher valence scores than those aged 30–34 while watching short videos that induced sadness (*M* = 2.72, *SD* = 0.61 versus *M* = 2.08, *SD* = 0.77; *p* < 0.001). The participants aged 25–29 gave significantly higher valence scores than those aged 20–24 while watching short videos that induced disgust (*M* = 2.85, *SD* = 0.84 versus *M* = 2.28, *SD* = 0.81; *p* < 0.001), fear (*M* = 2.80, *SD* = 0.98 versus *M* = 1.89, *SD* = 0.59; *p* < 0.001) and amusement (*M* = 6.56, SD = 1.03 versus *M* = 6.08, *SD* = 0.74; *p* = 0.014). The participants aged 25–29 gave significantly higher valence scores than those aged 30–34 while watching short videos that induced fear (*M* = 2.80, *SD* = 0.98 versus *M* = 2.05, *SD* = 0.66; *p* < 0.001). The participants aged 30–34 gave significantly higher valence scores than those aged 20–24 while watching short videos that induced disgust (*M* = 2.64, *SD* = 0.75 versus *M* = 2.28, *SD* = 0.81; *p* = 0.030) and amusement (*M* = 6.80, *SD* = 0.93 versus *M* = 6.08, *SD* = 0.74; *p* < 0.001). As shown in Fig 3B, among the female participants, the participants aged 20–24 gave significantly higher valence scores than those aged 25–29 while watching short videos that induced tenderness (*M* = 6.86, *SD* = 0.93 versus *M* = 6.22, *SD* = 0.85; *p* < 0.001). The participants aged 25–29 gave significantly higher valence scores than those aged 20–24 while watching short videos that induced sadness (*M* = 2.34, *SD* = 0.81 versus *M* = 1.92, *SD* = 0.65; *p* = 0.004) and amusement (*M* = 6.81, *SD* = 0.77 versus *M* = 6.34, *SD* = 0.90; *p* = 0.018). The participants aged 30–34 gave significantly higher valence scores than those aged 20–24 while watching short videos that induced amusement (*M* = 6.77, *SD* = 1.04 versus *M* = 6.34, *SD* = 0.90; *p* = 0.034). Secondly, the simple-simple effect analysis of gender on age and emotion category was also carried out. As shown in Fig 4A, among the participants aged 20–24, the male had higher valence scores than female while watching short videos that induced disgust (*M* = 2.28, *SD* = 0.81 versus *M* = 1.89, *SD* = 0.65; *p* = 0.005) and sadness (*M* = 2.72, *SD* = 0.61 versus *M* = 1.92, *SD* = 0.65; *p* < 0.001). While the female had higher valence scores than male when inducing anger (*M* = 2.88, *SD* = 0.82 versus *M* = 2.40, *SD* = 0.44; *p* = 0.001), fear (*M* = 2.43, *SD* = 0.82 versus *M* = 1.89, *SD* = 0.59; *p* < 0.001), neutrality (*M* = 5.12, *SD* = 0.60 versus *M* = 4.75, *SD* = 0.70; *p* = 0.020) and tenderness (*M* = 6.86, *SD* = 0.93 versus *M* = 6.35, *SD* = 0.90; *p* = 0.001). As shown in Fig 4B, among the participants aged 25–29, the male had higher valence scores than female while watching short videos that induced disgust (*M* = 2.85, *SD* = 0.84 versus *M* = 1.99, *SD* = 0.64; *p* < 0.001). While the female had higher valence scores than male when inducing sadness (*M* = 2.34, *SD* = 0.81 versus *M* = 2.03, *SD* = 0.68; *p* = 0.017). As shown in Fig 4C, among the participants aged 30–34, the male had higher valence scores than female while watching short videos that induced disgust (*M* = 2.64, *SD* = 0.75 versus *M* = 2.07, *SD* = 0.79; *p* < 0.001). While the female had higher valence scores than male when inducing anger (*M* = 2.87, *SD* = 0.86 versus *M* = 2.55, *SD* = 0.93; *p* = 0.024), fear (*M* = 2.50, *SD* = 0.87 versus *M* = 2.05, *SD* = 0.66; *p* = 0.004) and tenderness (*M* = 6.59, *SD* = 0.71 versus *M* = 6.09, *SD* = 0.62; *p* = 0.001).

For the factor of gender, a significant main effect was also found (*F*_(1,354)_ = 13.12, *p* < 0.001, *η*^2^ = 0.04). The *post-hoc* test indicated that the valence scores of the female were significantly higher than male (*M* = 4.29, *SD* = 2.16 versus *M* = 4.17, *SD* = 2.02; *p* < 0.001). In addition, there were no significant differences revealed in the main effect of age (*F*_(2,354)_ = 2.54, *p* = 0.080, *η*^2^ = 0.01), as well as the interaction effect of age by gender (*F*_(2,354)_ = 1.47, *p* = 0.231, *η*^2^ = 0.01).

#### Arousal effects

For each emotion category scored by participants of different ages and genders, the descriptive statistical results on the arousal dimension are shown in Table 2. The values of *M* and *SD* were calculated to evaluate the arousal degree of participants elicited by short videos.

**Table 2:** The descriptive statistical results on the arousal dimension.

		Disgust	Anger	Fear	Sadness	Neutrality	Tenderness	Amusement	Joy	Total
20–24	Male	5.98 (0.73)	5.83 (1.12)	6.26 (1.46)	6.13 (0.84)	2.09 (0.64)	6.25 (1.45)	6.14 (1.02)	6.57 (1.13)	5.66 (1.74)
	Female	6.52 (0.91)	5.92 (1.01)	6.11 (0.93)	6.31 (0.91)	2.34 (0.88)	6.93 (1.29)	6.18 (1.06)	6.24 (1.20)	5.82 (1.69)
	Total	6.25 (0.87)	5.87 (1.06)	6.18 (1.22)	6.22 (0.87)	2.22 (0.78)	6.59 (1.41)	6.16 (1.04)	6.40 (1.17)	5.74 (1.72)
25–29	Male	6.64 (0.88)	6.00 (1.25)	6.12 (0.84)	5.99 (0.74)	2.11 (0.60)	6.06 (1.49)	6.04 (1.10)	5.93 (1.43)	5.61 (1.72)
	Female	6.63 (1.01)	6.33 (1.45)	6.46 (1.12)	6.44 (1.09)	2.37 (0.84)	6.83 (1.12)	6.24 (1.12)	6.42 (1.14)	5.97 (1.77)
	Total	6.63 (0.94)	6.17 (1.36)	6.29 (1.00)	6.22 (0.95)	2.24 (0.74)	6.45 (1.37)	6.14 (1.11)	6.17 (1.31)	5.79 (1.75)
30–34	Male	6.29 (0.80)	6.32 (1.02)	6.08 (1.04)	5.99 (0.92)	2.04 (0.65)	6.08 (1.43)	6.05 (1.24)	5.96 (1.42)	5.60 (1.74)
	Female	6.21 (0.77)	6.06 (1.35)	6.21 (1.13)	5.98 (0.92)	2.30 (0.88)	6.65 (1.17)	6.46 (1.17)	6.43 (1.14)	5.79 (1.71)
	Total	6.25 (0.78)	6.19 (1.20)	6.14 (1.08)	5.98 (0.92)	2.17 (0.78)	6.36 (1.33)	6.25 (1.21)	6.19 (1.30)	5.69 (1.72)
Total	Male	6.30 (0.85)	6.05 (1.14)	6.15 (1.14)	6.04 (0.83)	2.08 (0.63)	6.13 (1.45)	6.08 (1.12)	6.15 (1.36)	5.62 (1.73)
	Female	6.45 (0.92)	6.11 (1.29)	6.26 (1.07)	6.24 (0.99)	2.34 (0.86)	6.80 (1.19)	6.29 (1.12)	6.36 (1.16)	5.86 (1.72)
	Total	6.38 (0.88)	6.08 (1.22)	6.21 (1.10)	6.14 (0.92)	2.21 (0.76)	6.47 (1.37)	6.18 (1.12)	6.26 (1.27)	5.74 (1.73)

**Fig 5:**
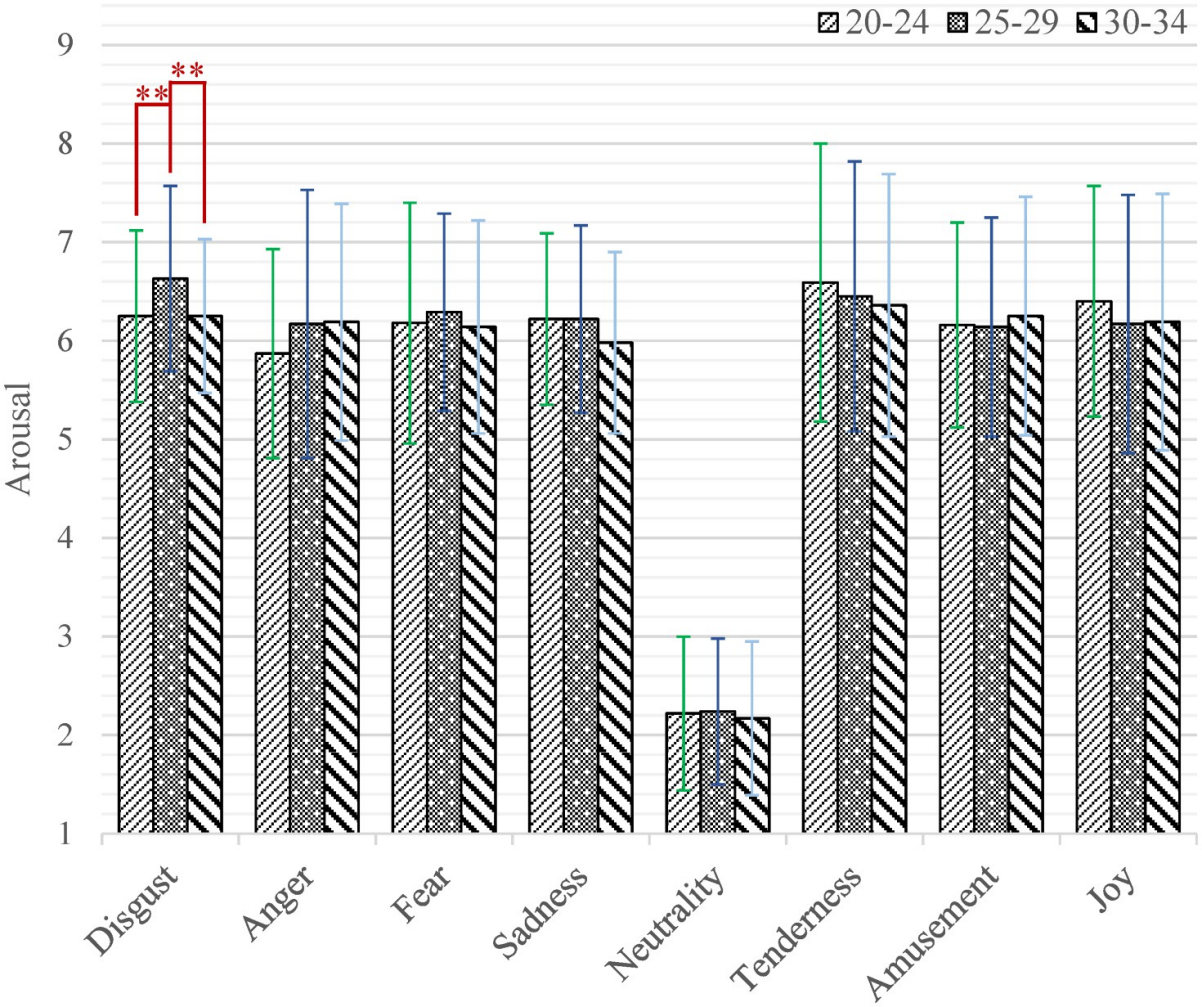
The arousal scores of each category of emotion-inducing short videos among participants with different ages. Statistical significance: *p < 0.05, **p < 0.01, ***p < 0.001. Unless marked with the asterisk, no significant differences between these groups were found.

When processing the three-way mixed ANOVA on the arousal dimension, there was a significant main effect of emotion category (*F*_(7,2478)_ = 634.75, *p* < 0.001, *η*^2^ = 0.64). The *post-hoc* test indicated that the arousal scores of neutral short videos were significantly lower than those of positive and negative ones (all *p* < 0.001). Furthermore, the short videos eliciting disgust were rated as higher arousal scores than those inducing anger (*M* = 6.38, *SD* = 0.88 versus *M* = 6.08, SD = 1.22; *p* = 0.005) and sadness (*M* = 6.38, *SD* = 0.88 versus *M* = 6.14, *SD* = 0.92; *p* = 0.010). The short videos eliciting tenderness were rated as higher arousal scores than those inducing anger (*M* = 6.47, *SD* = 1.37 versus *M* = 6.08, *SD* = 1.22; *p* < 0.001) and sadness (*M* = 6.47, *SD* = 1.37 versus *M* = 6.14, *SD* = 0.92; *p* = 0.005). The interaction effect of emotion category by age (*F*_(14,2478)_ = 1.76, *p* = 0.039, *η*^2^ = 0.10) was significant. Further, the results of simple effect analysis are shown in Fig 5. While watching short videos that induced disgust, the participants aged 25–29 showed significantly higher arousal scores than those aged 20–24 (*M* = 6.63, *SD* = 0.94 versus *M* = 6.25, *SD* = 0.87; *p* = 0.002) and 30–34 (*M* = 6.63, *SD* = 0.94 versus *M* = 6.25, *SD* = 0.78; *p* = 0.002).

**Fig 6:**
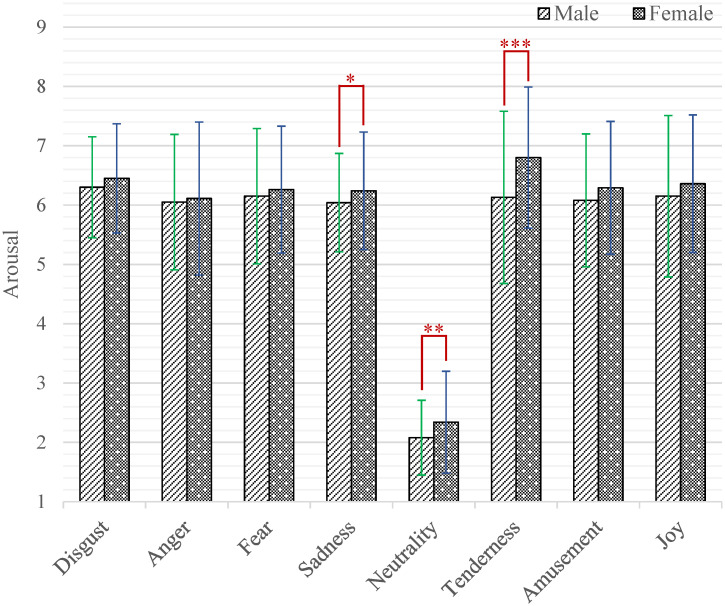
The arousal scores of each category of emotion-inducing short videos between male and female. Statistical significance: *p < 0.05, **p < 0.01, ***p < 0.001. Unless marked with the asterisk, no significant differences between these groups were found.

At the same time, the interaction effect of emotion category by gender (*F*_(7,2478)_ = 2.76, *p* = 0.010, *η*^2^ = 0.008) was also significant. Further, the results of simple effect analysis are shown in Fig 6. The female participants had higher arousal scores than male while watching short videos that induced sadness (*M* = 6.24, *SD* = 0.99 versus *M* = 6.04, *SD* = 0.83; *p* = 0.033), neutrality (*M* = 2.34, *SD* = 0.86 versus *M* = 2.08, *SD* = 0.63; *p* = 0.002) and tenderness (*M* = 6.80, *SD* = 1.19 versus *M* = 6.13, *SD* = 1.45; *p* < 0.001).

**Fig 7:**
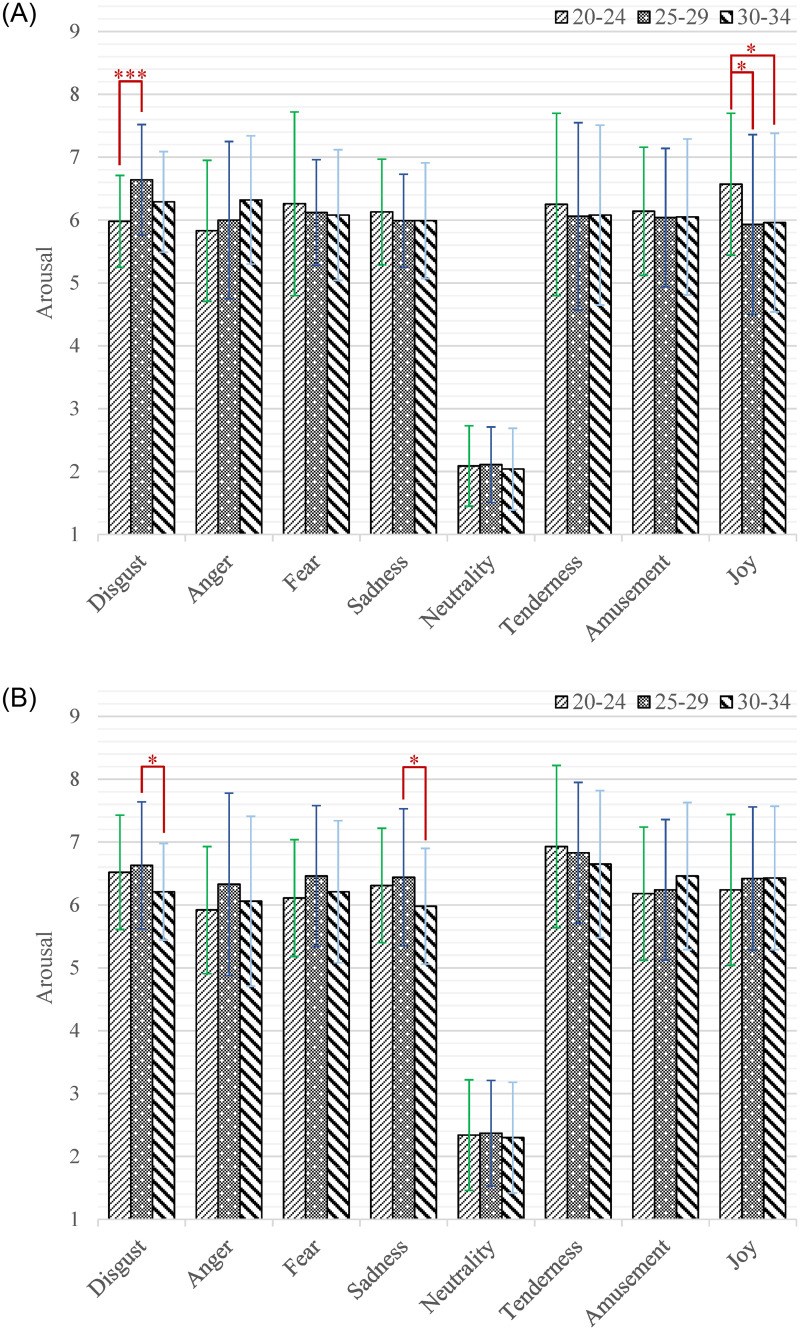
The arousal scores of each category of emotion-inducing short videos among participants with different ages. A: Male. B: Female. Statistical significance: *p < 0.05, **p < 0.01, ***p < 0.001. Unless marked with the asterisk, no significant differences between these groups were found.

**Fig 8:**
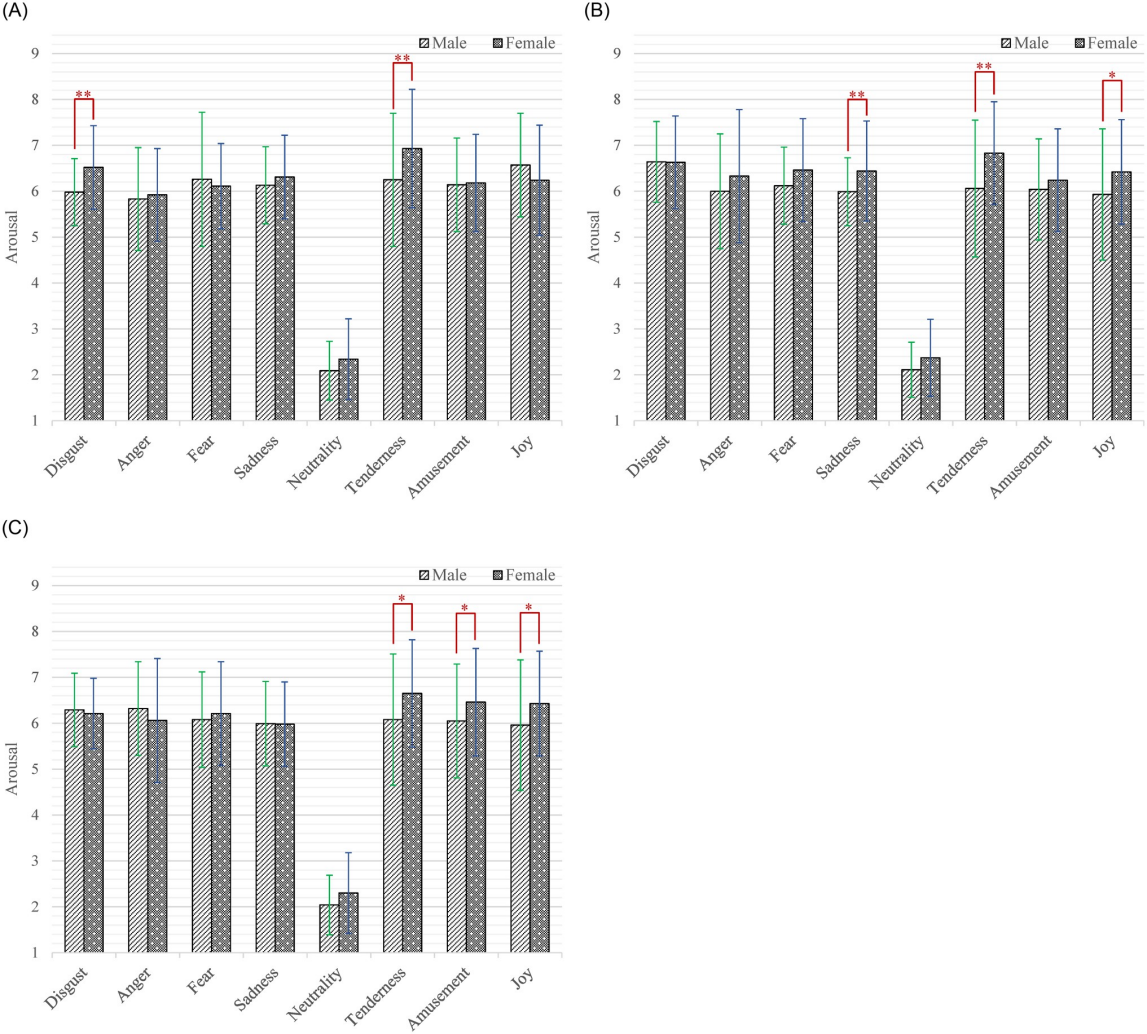
The arousal scores of each category of emotion-inducing short videos between male and female. A: Age = 20–24. B: Age = 25–29. C: Age = 30–34. Statistical significance: *p < 0.05, **p < 0.01, ***p < 0.001. Unless marked with the asterisk, no significant differences between these groups were found.

Moreover, the interaction effect of emotion category by age by gender (*F*_(14,2478)_ = 1.81, *p* = 0.038, *η*^2^ = 0.10) was significant. According to our research interests, the simple-simple effect analysis of age on gender and emotion category was firstly conducted. As shown in Fig 7A, among the male participants, the participants aged 20–24 gave significantly higher arousal scores than those aged 25–29 while watching short videos that induced joy (*M* = 6.57, *SD* = 1.13 versus *M* = 5.93, *SD* = 1.43; *p* = 0.015). The participants aged 20–24 gave significantly higher arousal scores than those aged 30–34 while watching short videos that induced joy (*M* = 6.57, *SD* = 1.13 versus *M* = 5.96, *SD* = 1.42; *p* = 0.025). The participants aged 25–29 gave significantly higher arousal scores than those aged 20–24 while watching short videos that induced disgust (*M* = 6.64, *SD* = 0.88 versus *M* = 5.98, *SD* = 0.73; *p* < 0.001). As shown in Fig 7B, among the female participants, the participants aged 25–29 gave significantly higher arousal scores than those aged 30–34 while watching short videos that induced disgust (*M* = 6.63, *SD* = 1.01 versus *M* = 6.21, *SD* = 0.77; *p* = 0.023) and sadness (*M* = 6.44, *SD* = 1.09 versus *M* = 5.98, *SD* = 0.92; *p* = 0.017). Then the simple-simple effect analysis of gender on age and emotion category was also performed. As shown in Fig 8A, among the participants aged 20–24, the female had higher arousal scores than male while watching short videos that induced disgust (*M* = 6.52, *SD* = 0.91 versus *M* = 5.98, *SD* = 0.73; *p* = 0.001) and tenderness (*M* = 6.93, *SD* = 1.29 versus *M* = 6.25, *SD* = 1.45; *p* = 0.005). As shown in Fig 8B, among the participants aged 25–29, the female had higher arousal scores than male while watching short videos that induced sadness (*M* = 6.44, *SD* = 1.09 versus *M* = 5.99, *SD* = 0.74; *p* = 0.007), tenderness (*M* = 6.83, *SD* = 1.12 versus *M* = 6.06, *SD* = 1.49; *p* = 0.002) and joy (*M* = 6.42, *SD* = 1.14 versus *M* = 5.93, *SD* = 1.43; *p* = 0.030). As shown in Fig 8C, among the participants aged 30–34, the female had higher arousal scores than male while watching short videos that induced tenderness (*M* = 6.65, *SD* = 1.17 versus *M* = 6.08, *SD* = 1.43; *p* = 0.020), amusement (*M* = 6.46, *SD* = 1.17 versus *M* = 6.05, *SD* = 1.24; *p* = 0.046) and joy (*M* = 6.43, *SD* = 1.14 versus *M* = 5.96, *SD* = 1.42; *p* = 0.043).

As for the factor of gender, a significant main effect was also found (*F*_(1,354)_ = 31.32, *p* < 0.001, *η*^2^ = 0.08). The *post-hoc* test indicated that the arousal score of the female were significantly higher than male (*M* = 5.86, *SD* = 1.72 versus *M* = 5.62, *SD* = 1.73; *p* < 0.001). Moreover, there were no significant differences presented in the main effect of age (*F*_(2,354)_ = 1.68, *p* = 0.188, *η*^2^ = 0.01), as well as the interaction effect of age by gender (*F*_(2,354)_ = 2.07, *p* = 0.127, *η*^2^ = 0.01).

#### Selected short videos

Through the analysis on valence and arousal effects, we found that when watching emotional short videos, the emotional responses of participants did differ by age and gender. Based on this finding, we have reasons to believe that targeting different emotional short videos for participants of different ages and genders will contribute to better emotion elicitation effects. In the experiment, we used an objective criterion of success index [5] to select effective short videos. For each short video, its success index is obtained by summing the z-scores of hit rate and intensity. Therein, the hit rate is measured as the proportion of participants who rated the target emotion at least one point higher than other emotion categories, and the intensity is the mean rating of target emotion [45]. Both the hit rate and intensity can be calculated by the results of the emotional evaluation scale rated by 360 participants.

Considering the existing EEG based emotion recognition algorithms have shown considerable performance in solving the two-category and three-category tasks, in order to further validate our database through EEG signals in the subsequent experiment, here we combined the original eight emotional categories into three emotional states and built a database of Chinese emotional short videos based on age and gender differences containing three valence states, i.e., positive, neutral and negative. Guided by the success index, we totally selected 54 short videos (duration: 91–229s, mean = 160.04s) as emotion elicitation materials for 6 groups of participants, including male and female respectively aged in 20–24, 25–29 and 30–34. Each group of participants corresponded to 9 short videos, specifically involving 3 positive, 3 neutral and 3 negative stimuli. In order to facilitate the use of our emotional short video database, we also developed the naming rules for short video files. The name of a short video file consists of four parts, i.e., “valence (positive, neutral, negative)_gender (1=“male”, 2= “female”)_age (1=“20–24 years”, 2=“25–29 years”, 3=“30–34 years”)_number (1, 2, 3).MP4”. For example, the file named “positive_2_3_1.MP4” denotes a short video used to induce positive emotion for females aged 30–34, and its number is 1. Due to the copyright issues, we are not able to provide the actual short videos, but the download links are included in “S1 Video” of the supporting information.

## Experiment 2

The purpose of Experiment 2 is to capture the EEG signals and subjective experiences of participants with different ages and genders when watching different emotional stimuli, 1) to verify the reliability of short videos as audio-visual stimulus materials for eliciting emotions compared with film clips; 2) to validate the effectiveness of targeted delivery of specific emotional short videos to different participant groups. Here we chose EEG to validate the selected 54 video clips, since it can directly record the changes in scalp potentials and reflect emotions more objectively and accurately compared with other physiological signals. In this experiment, the emotion recognition results based on EEG can also provide the objective support for subjective experience ratings.

### Methods

#### Ethics

Ethics was the same as Experiment 1.

#### Materials

In the Experiment 1, we totally selected 54 emotional short videos for 6 groups of participants with different ages and genders. For each group, 9 short videos were provided including 3 positive, 3 neutral and 3 negative stimuli. To make a fair comparison, we also selected 9 film clips (duration: 41–114 seconds, mean = 67.78 seconds) from the Chinese film database developed in [45], which includes 22 film clips (duration: 41–166 seconds, mean = 82.50 seconds) for eliciting eight categories of emotions, i.e., four negative emotions (disgust, anger, fear and sadness), neutrality and three positive emotions (tenderness, amusement and joy). More specifically, three positive (Singing When We Are Young, Just Another Pandora’s Box, Hear Me), three neutral (Raise the Red Lantern, Black Coal Thin Ice, Space Millennium) and three negative film clips (City of Life and Death, Bodyguards and Assassins, The Chrysalis) were picked out according to the rank of success index. Similar to the emotional short videos, the 9 film clips were renamed in the format of “valence (positive, neutral, negative)_film_number (1, 2, 3).MP4”. For example, the file named “positive_film_1.MP4” stands for the film clip “Singing When We Are Young”.

#### Participants

Since we needed to collect the EEG signals of participants, two additional conditions were added besides the participant recruitment requirements given in Experiment 1: no head wound and no hair dye or perm within one month. To ensure the independence of Experiment 1 and Experiment 2, the participants recruited in this experiment was different with those in Experiment 1. We totally received 412 applications, of which 100 volunteers were selected based on the evaluation results of EPQ-RSC and recruitment requirements. Before the experiment, the participants were reminded to get quality sleep, wash hair in advance, and avoid the intake of stimulating foods (e.g., tobacco, alcohol, coffee). As in Experiment 1, each participant was informed of the experimental procedure and signed an informed consent form. During the acquisition of EEG signals, we further excluded 19 participants because they could not adjust to a relaxed state. The final samples were composed of 81 participants (age range 20–24: 36 males and 20 females; 25–29: 8 males and 7 females; 30–34: 5 males and 5 females). Each participant received 150 RMB as payment after completing the whole experiment.

#### Measures

The measures were the same as those taken in Experiment 1. Specifically for the integrity of subjective assessment, three dimensions were added to the Self-assessment 9-point scale in this experiment. They are namely dominance (from being totally controlled to having strong control power), liking (from very disliked to very liked) and familiarity (from very unfamiliar to very familiar).

#### Procedure

**Fig 9:**
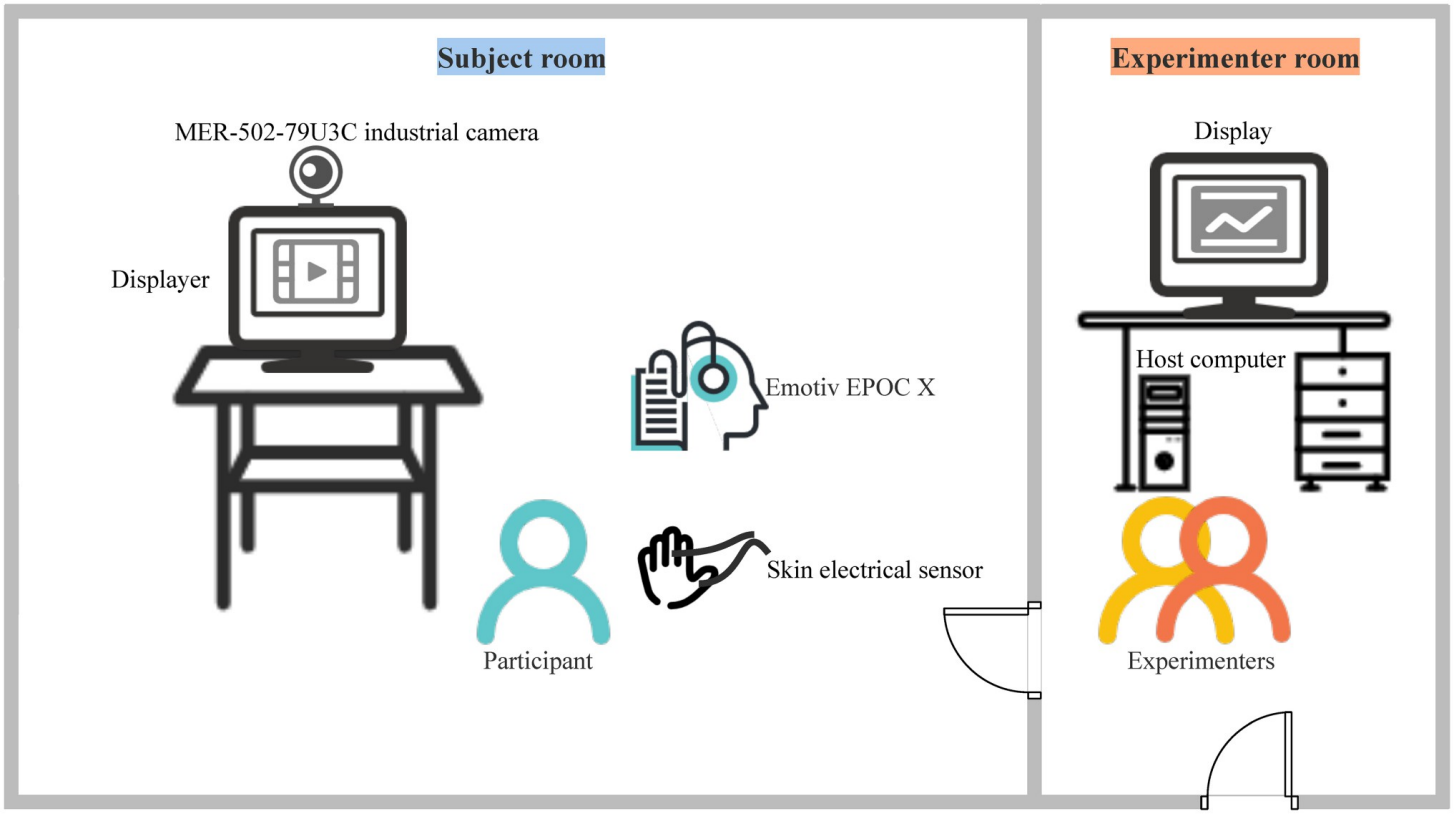
The experimental environment.

**Fig 10:**
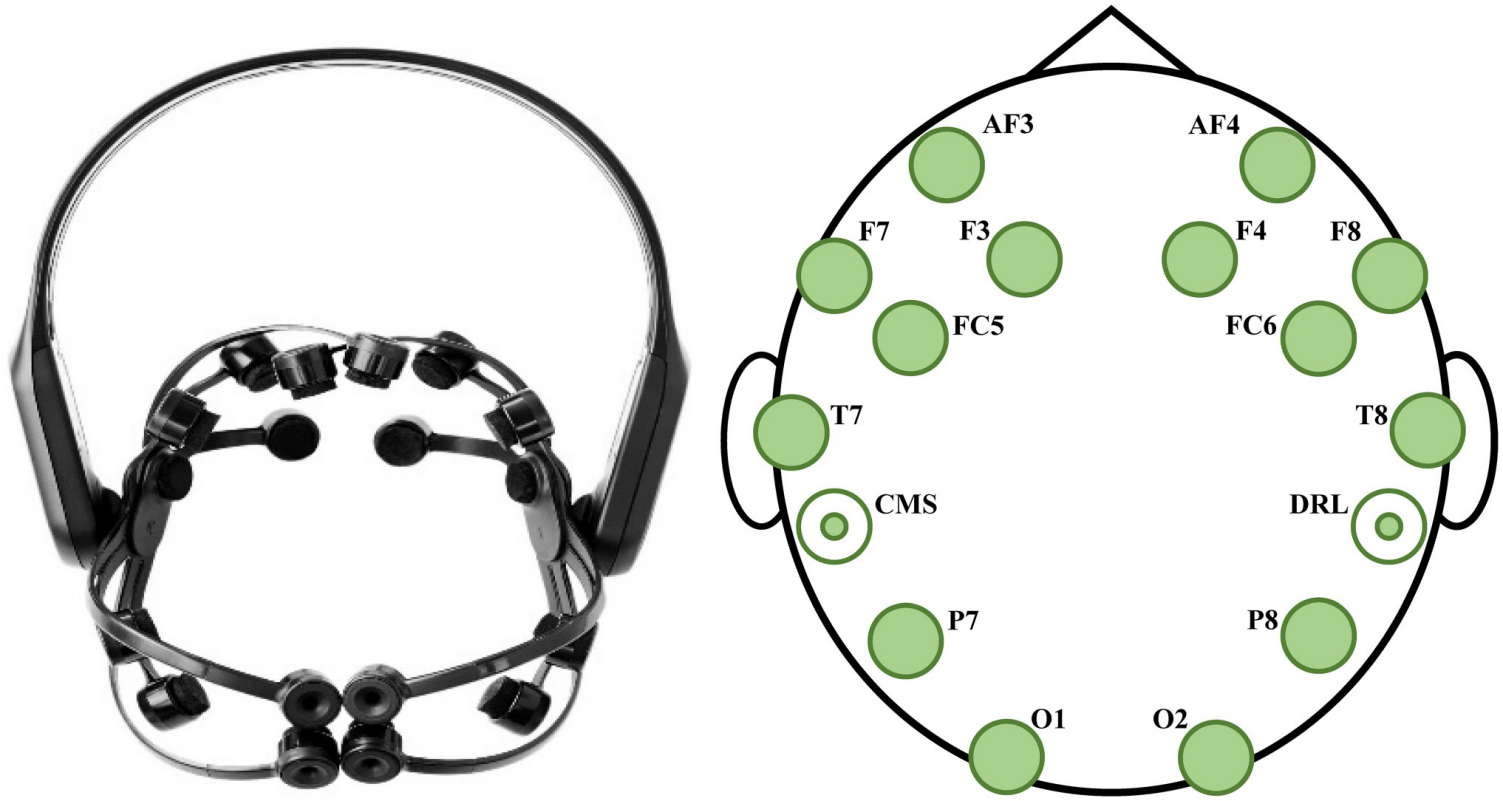
The Emotiv EPOC X and its electrode distribution.

The experimental environment consists of the subject room and the experimenter room. As shown in Fig 9, the EEG signal acquisition experiment was conducted in a quiet, bright subject room. The stimuli were presented by a 24.5-inch displayer with a refresh rate of 165 Hz. The EEG signals were acquired by Emotiv EPOC X, which is a wireless portable EEG collecting device with 14 channels (i.e., AF3, F7, F3, FC5, T7, P7, O1, O2, P8, T8, FC6, F4, F8 and AF4 in the international 10–20 system), 0.2–43Hz bandwidth and 256Hz sampling frequency. The Emotiv EPOC X and its electrode distribution are shown in Fig 10. The EEG data were recorded by EmotivPRO software. At the same time, we used the MER-502–79U3C industrial camera to capture the face videos and skin electrical sensor to obtain GSR data from left hand of the participant. In order to avoid the interference of electromagnetic devices on the EEG signals, the displayer in the subject room was connected to the host computer in a wired way. The host computer was placed in the experimenter room, and the experimenters could observe the situations of equipment connection and participant status in real time through the displayer in the experimenter room.

Before the start of the formal experiment, the experimenter first introduced the experimental procedure and subjective evaluation scales in detail. And then, two experimenters helped the participant to wear the EEG cap and skin electrical sensor. The participant was reminded to adjust the seat to a comfortable state according to his viewing habits to ensure that he could see the screen clearly. Subsequently, the participant got familiar with the experimental procedure through a practice (one trial). The practice consisted of five steps, including baseline recording, watching the video stimulus, filling in subjective evaluation scales, completing calculation questions and taking a break. During a 15-second baseline recording, the participant was required to remain relaxed, blink as infrequently as possible, and look at a fixation cross “+” on the screen. Then a video stimulus was displayed and the participant was asked to stay as still as possible and blink as infrequently as possible when watching the stimulus. After that, the participant filled in the subjective evaluation scales based on his immediate true feelings. To eliminate the effect of the previous stimulus, the participant was asked to complete two simple calculation questions within ten as a distraction. Next, a 30-second period of rest was taken, during which a blank screen was displayed and the participant was asked to clear his brain of all thoughts, feelings and memories as much as possible. When the participant was ready to start the formal experiment, two experimenters left the subject room.

**Fig 11:**
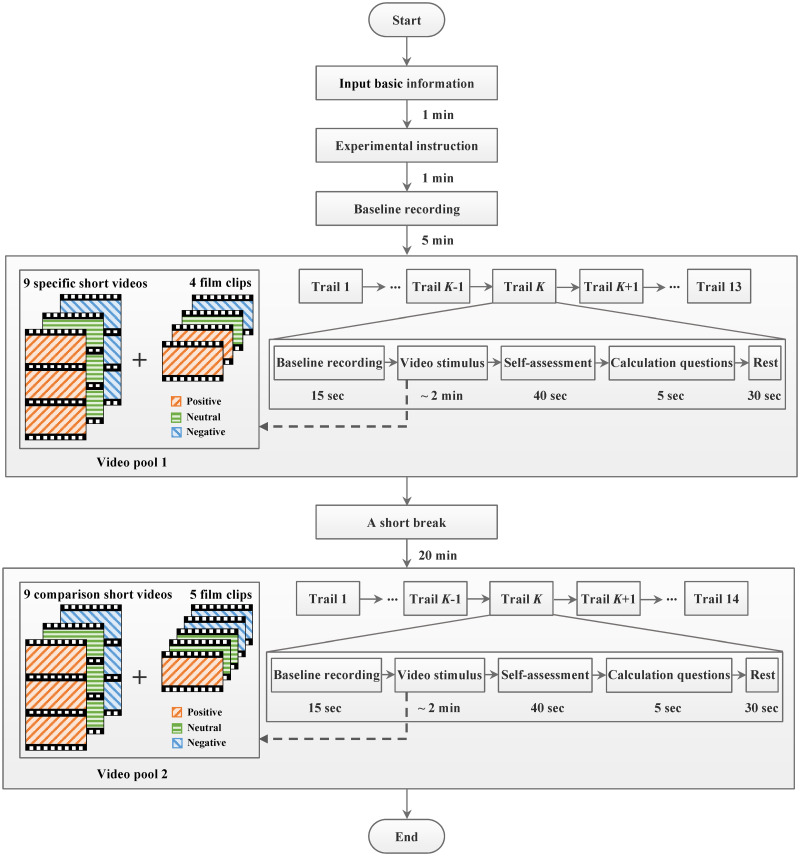
The flowchart of EEG signal acquisition experiment.

**Fig 12:**
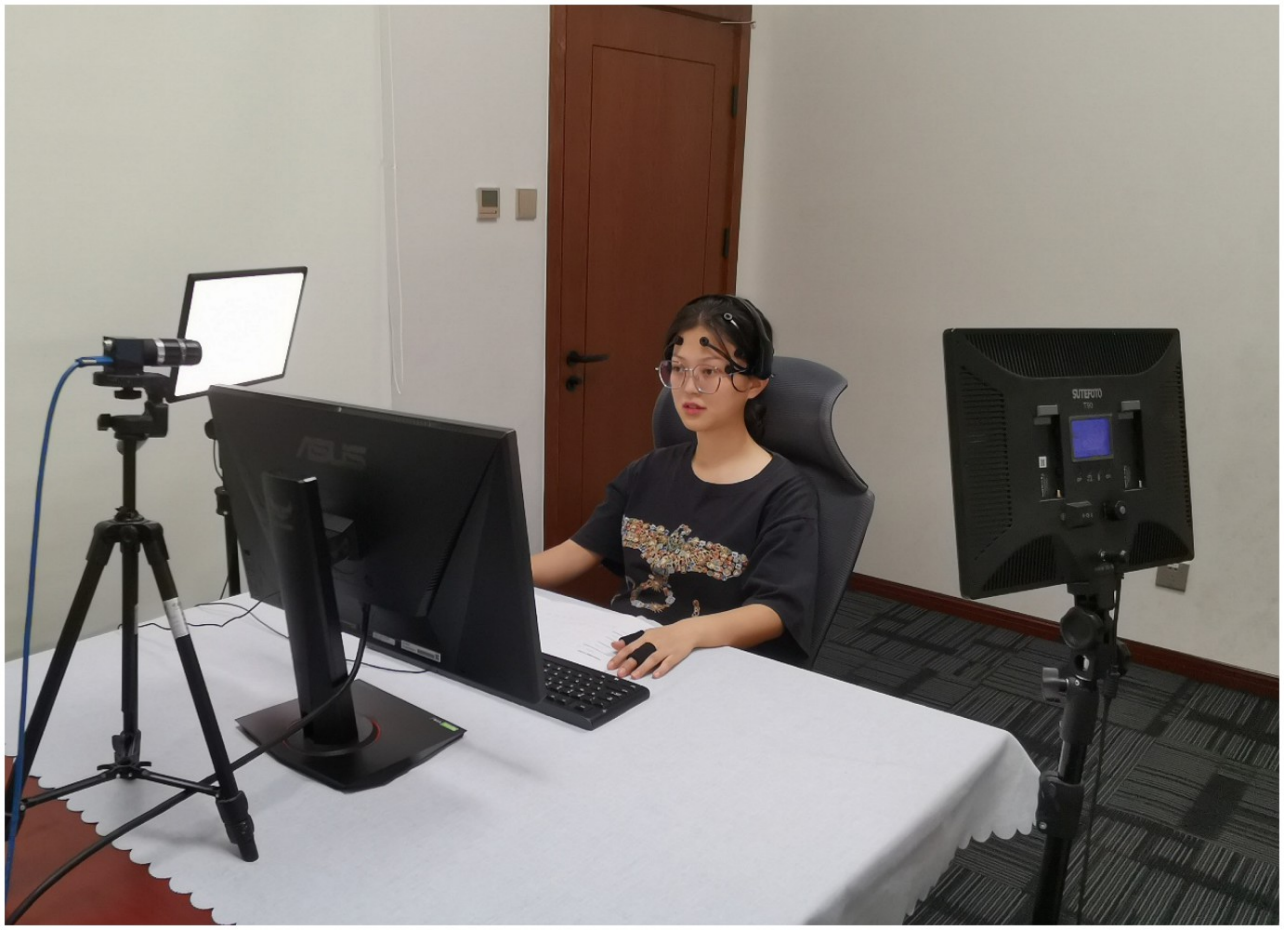
A participant shortly before the experiment.

In order to reduce the influence of human biological rhythms, the experiments were carried out during the daytime. Concretely, the experimental periods were 9:00–11:30 am and 3:00–5:30 pm. Fig 11 shows the flowchart of EEG signal acquisition experiment. When the participant clicked the start button on the screen, he was asked to fill in basic information (including age, gender, profession, education and major), read the experiment description and complete a 5-minute baseline recording with opening or closing eyes alternately every 15 seconds. It can be seen from Fig 11 that, the whole experiment includes two blocks with a total of 27 trails. In the first block, the participant watched a total of 13 video stimuli, consisting of 9 specific short videos (3 positive, 3 neutral and 3 negative) tailored to his age and gender and 4 film clips from the selected 9 Chinese film clips (2 positive, 1 neutral and 1 negative). While in the second block, the participant was required to watch a total of 14 video stimuli, including 9 comparison short videos (3 positive, 3 neutral and 3 negative) designed for analyzing the differences in age and gender and other 5 film clips (1 positive, 2 neutral and 2 negative). Particularly, no more than three video stimuli with the same valence state were shown continuously. To avoid fatigue, there was a short break of about 20-minute between the two blocks. During the experiment, the participant could terminate the experiment immediately if he feels any discomfort. Fig 12 shows a participant shortly before the start of the experiment.

In the EEG signal acquisition experiment, we considered the effects of age and gender differences on emotion elicitation from the following four aspects. For analyzing the effect of gender difference on emotion elicitation, we selected two groups of participants with 20 males and 20 females both aged 20–24. In the aspect of age difference, two groups of participants were chosen, namely 8 males aged 20–24 and 5 males aged 30–34. For analyzing the combined effect of age and gender differences, we picked out two groups of participants with 8 males aged 20–24 and 5 females aged 30–34. For the two groups in the above analysis, the specific short videos of one group were taken as the comparison ones of the other group. For analyzing the effect of specific short videos designed for participants of different ages and genders, two groups of participants were selected, including 8 males and 7 females both aged 25–29. For each group, the comparison short videos were randomly selected from those designed for other groups.

#### Data processing

The processing of the raw EEG data includes preprocessing and feature extraction. In the preprocessing phase, a bandpass filter was employed to retain the EEG data in the frequency interval of 0.1–50Hz, and a notch filter was also used for denoising with the frequency being 50Hz. Then we manually removed the artifacts to avoid the significant electromyogram and electro-oculogram due to muscle contraction, blinking or eye movement. For the EEG data of each participant, no more than 10 percent of the original data were removed. In the feature extraction phase, the EEG data were sliced into 1-second segments without overlapping, and the differential entropy (DE) features [48] were extracted in five frequency bands, i.e., *δ* (1–4Hz), *θ* (4–8Hz), *α* (8–12Hz), *β* (13–30Hz) and *γ* (31–45Hz).

### Results

#### EEG emotion recognition results

**Fig 13:**
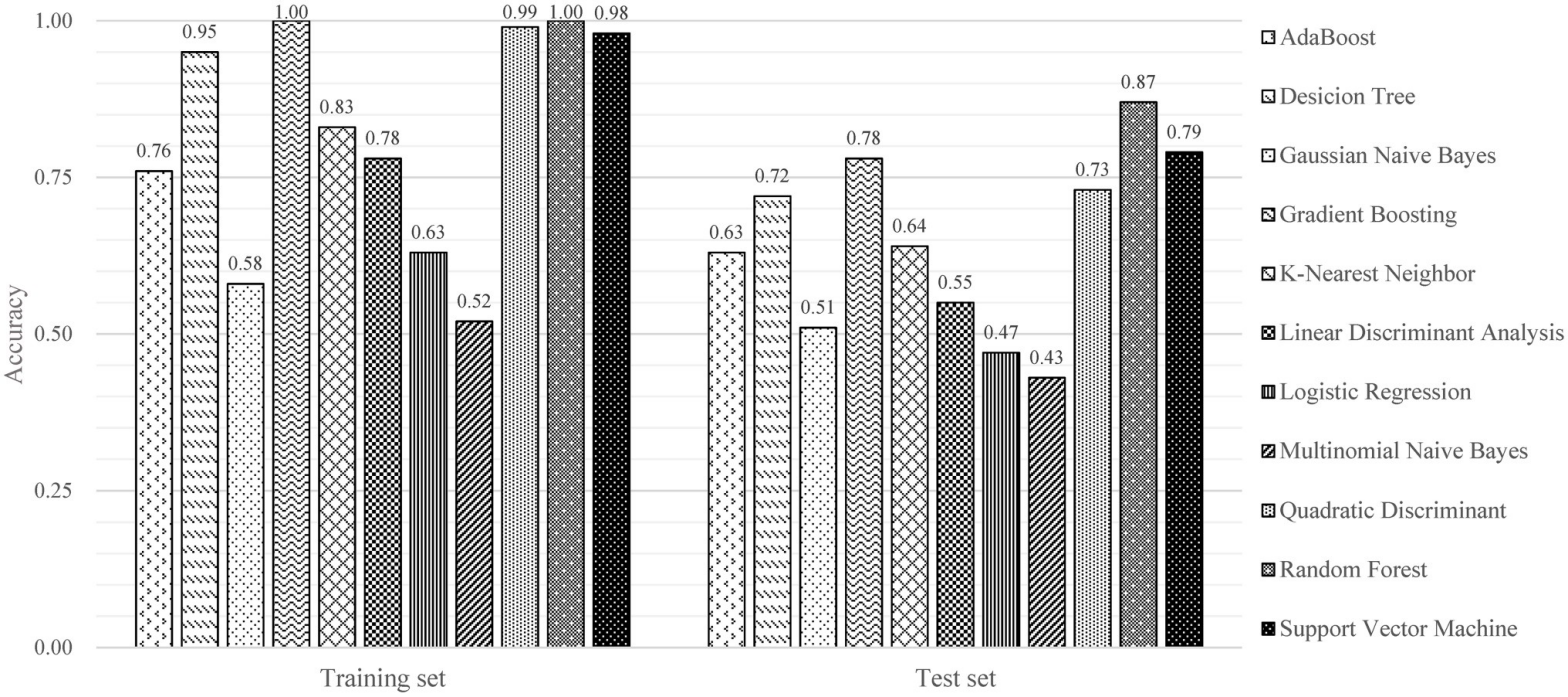
The emotion recognition results of 11 commonly used classifiers.

In this subsection, we used the EEG signals to perform the emotion recognition task, and then the elicitation effects of emotional stimuli can be evaluated by the recognition accuracy. Since the performance of the classifier is directly related to the accuracy of emotion recognition results, we firstly compared the performance of 11 classifiers that are commonly used in the machine learning methods. In the experiment, the EEG data from all 81 participants were divided into training set and test set in the ratio of 8:2. The input of these classifiers was the DE features of EEG signals, and the output was the three-category results of emotions (positive, neutral and negative). The accuracies of different classifiers for the EEG emotion recognition are given in Fig 13. It can be seen from Fig 13 that, the algorithm of Random Forest achieves the highest accuracy of 86.59% among all 11 classifiers. Therefore, we selected the Random Forest algorithm as the classifier of the EEG emotion recognition task for further study.

Table 3 shows the EEG emotion recognition results of different participant groups elicited by different video stimuli. The values in bold indicate the highest accuracy for each group of participants. It can be seen from Table 3 that for both male and female participants aged 20–24, the accuracies of EEG signals elicited by specific short videos are higher than those by comparison ones (male: 96.12% versus 91.22%; female: 87.28% versus 83.87%). This indicates that the gender difference does exist when watching emotional short videos, and thus the specific short videos designed for participants of different genders exhibit better emotion elicitation effects. Similar to the analysis of gender difference, we can observe from Table 3 that the age difference does also exist, so does the combined effect of age and gender differences. For different groups, the accuracies of EEG signals elicited by specific short videos are higher than those by comparison ones. In addition, for both male and female participants aged 25–29, the accuracies of EEG signals elicited by specific short videos are also higher than those by the short videos randomly selected from those designed for other groups, validating the effectiveness of targeted delivery of specific emotional short videos to different participant groups. What’s more, both the specific and comparison short videos bring higher average accuracies than film clips (91.39%/86.37% versus 81.12%), which verifies the reliability of short videos as audio-visual stimuli for eliciting emotions compared with film clips.

**Table 3:** The EEG emotion recognition results of different participant groups elicited by different video stimuli.

Influence factor	Participants	Short videos (%)		Film clips (%)
		Specific	Comparison	
Gender	20–24 male	**96.12**	91.22	84.58
	20–24 female	**87.28**	83.87	76.82
Age	20–24 male	**96.57**	90.05	85.43
	30–34 male	**90.24**	87.32	81.67
Age_gender	20–24 male	**95.18**	89.81	86.25
	30–34 female	**89.41**	80.56	72.34
Specific_random	25–29 male	**92.18**	89.79	81.65
	25–29 female	**84.16**	78.33	80.19
Average accuracy		**91.39**	86.37	81.12

#### Subjective evaluation results

In this subsection, we also conducted the statistical analysis on the collected subjective evaluation results. Considering the space constraints, here we take the male and female groups aged 20–24 when studying gender difference as an example, and show the statistical analysis results of their subjective evaluation in Tables 4 and 5. In Table 4, the statistical analysis is performed from the aspects of intensity, hit rate and success index on the different emotional stimuli. For each group of participants, the success indices in bold represent the three highest values for each emotion category. For the positive emotion state, among three video stimuli with the highest success indices for male group, two are from the specific short videos and one is from the comparison ones. The three video stimuli with the highest success indices for female group all come from the specific short videos. In terms of neutral emotion category, the three video stimuli with the highest success indices for both male and female groups all come from the specific short videos. For the negative emotion category, the three video stimuli with the highest success indices for male groups all come from the specific short videos. Among three video stimuli with the highest success indices for female group, two are from the specific short videos and one is from the comparison ones. From the above analysis we can conclude that, of all 18 video stimuli with the highest success indices for three emotion categories, 16 are from the specific short videos and 2 are from the comparison ones. Therefore, it can also be verified that the emotion elicitation effects of short videos are better than those of film clips, and further the targeting delivery of specific emotional short videos makes sense to different participant groups. In addition, Table 4 also shows the comparisons between male and female groups on the intensity and hit rate for each video stimulus. It can be seen from Table 4 that there is the significant gender difference in the intensity of target emotion experienced for 10 short videos. This was due to the participants rating their emotions as more intense when watching the specific short videos than other ones. Moreover for the aspect of hit rate, no significant differences were found between the male and female groups aged 20–24. In addition, Table 5 presents the statistical analysis results on SAM scale of male and female groups aged 20–24, including valence, arousal, liking, dominance and familiarity. It can be seen from Table 5 that, the significant differences among the specific short videos in terms of the ratings of valence and arousal reflect the successful elicitation of the targeted emotions. Furthermore, we found that the participants generally scored their liking and familiarity with specific short videos higher than comparison short videos and film clips.

**Table 4:** The statistical analysis results on emotional evaluation scale of male and female groups aged 20–24.

	Intensity					Hit Rate (%)			Success Index	
	Male		Female		*T*-value	Male	Female	Chi-sq.	Male	Female
Stimulus Name	*M*	*SD*	*M*	*SD*		Freq.	Freq.		*M*	*M*
positive_1_1_1	7.55	1.20	6.55	1.83	1.99	85.00	65.00	1.20	**1.34**	-2.47
positive_1_1_2	7.80	1.21	6.40	1.11	3.71***	80.00	70.00	0.13	1.05	-2.32
positive_1_1_3	7.80	1.03	6.70	1.42	2.74**	90.00	85.00	0.00	**2.40**	-0.61
positive_2_1_1	6.80	1.96	7.80	0.75	-2.07*	80.00	100.00	2.50	-0.48	**2.43**
positive_2_1_2	7.35	1.15	7.75	0.94	-1.17	90.00	100.00	0.53	**1.72**	**2.35**
positive_2_1_3	6.95	1.53	7.20	1.17	-0.57	80.00	100.00	2.50	-0.25	**1.43**
positive_film_1	5.65	2.22	6.20	1.63	-0.87	75.00	90.00	0.96	-2.92	-1.04
positive_film_2	6.65	1.35	6.10	1.64	1.13	85.00	95.00	0.28	-0.03	-0.80
positive_film_3	6.60	1.24	7.20	1.63	-1.28	65.00	95.00	3.91*	-2.82	1.03
neutral_1_1_1	7.90	1.51	7.15	1.62	1.47	95.00	95.00	0.00	**2.21**	0.24
neutral_1_1_2	7.55	1.28	7.25	1.30	0.72	90.00	95.00	0.00	**1.41**	0.44
neutral_1_1_3	8.00	1.10	6.40	2.08	2.96**	100.00	95.00	0.00	**2.70**	-1.25
neutral_2_1_1	7.05	1.66	8.05	0.74	-2.40*	70.00	100.00	4.90*	-0.68	**2.60**
neutral_2_1_2	6.95	1.99	7.25	1.34	-0.55	80.00	100.00	2.50	-0.07	**1.00**
neutral_2_1_3	7.15	1.28	7.25	1.04	-0.26	85.00	100.00	1.44	0.55	**1.00**
neutral_film_1	6.15	2.29	6.30	0.95	-0.26	60.00	70.00	0.11	-2.53	-4.21
neutral_film_2	5.30	2.47	6.80	1.57	-2.23*	60.00	90.00	3.33	-3.58	-1.01
neutral_film_3	6.70	1.71	7.35	1.19	-1.36	85.00	100.00	1.44	-0.01	1.20
negative_1_1_1	7.85	0.96	7.65	1.06	0.61	100.00	95.00	0.00	**1.62**	-0.56
negative_1_1_2	8.75	0.54	7.90	0.77	3.96***	95.00	100.00	0.00	**2.07**	**1.94**
negative_1_1_3	7.70	1.10	6.90	1.26	2.08*	100.00	95.00	0.00	**1.48**	-2.05
negative_2_1_1	6.85	1.31	8.10	0.94	-3.37**	95.00	100.00	0.00	0.31	**2.34**
negative_2_1_2	7.85	1.46	8.25	1.04	-0.97	95.00	100.00	0.00	1.24	**2.64**
negative_2_1_3	7.10	1.14	7.55	0.97	-1.31	90.00	100.00	0.53	0.16	1.25
negative_film_1	4.85	2.50	7.20	1.78	-3.34**	60.00	95.00	5.16*	-4.20	-1.46
negative_film_2	6.00	2.07	7.00	1.48	-1.71	70.00	95.00	2.77	-2.37	-1.85
negative_film_3	7.00	1.41	6.80	1.17	0.48	85.00	95.00	0.28	-0.31	-2.25

Statistical significance:

*p < 0.05,

**p < 0.01,

***p < 0.001.

*T*-value was the paired-samples *t*-tests of intensities between the male and female. Chi-sq. was the chi-square tests of hit rates between the male and female. Freq. was the frequency of hit rates.

**Table 5:** The statistical analysis results on SAM scale of male and female groups aged 20–24.

	Valence		Arousal		Liking		Dominance		Familiarity	
Stimulus Name	Male	Female	Male	Female	Male	Female	Male	Female	Male	Female
positive_1_1_1	7.45(1.02)	6.50(0.97)	6.95(0.92)	6.20(1.50)	7.20(1.12)	5.53(1.02)	6.80(0.87)	5.95(0.92)	6.20(1.72)	3.85(1.56)
positive_1_1_2	7.50(1.16)	6.40(1.11)	6.70(0.95)	6.10(1.30)	6.65(1.39)	4.85(1.96)	6.45(1.13)	5.10(1.76)	5.00(2.47)	2.12(1.31)
positive_1_1_3	7.50(0.92)	6.80(0.87)	6.85(0.96)	6.00(0.63)	7.20(1.25)	5.95(1.43)	6.35(1.46)	5.65(1.28)	5.60(2.62)	4.85(1.49)
positive_2_1_1	6.65(0.85)	7.35(0.85)	6.30(1.05)	7.15(0.91)	6.40(1.24)	7.80(0.62)	6.15(1.01)	6.45(1.07)	3.55(1.86)	6.70(1.00)
positive_2_1_2	7.45(0.67)	7.08(0.83)	6.85(1.28)	6.80(0.81)	5.55(1.02)	6.85(1.06)	5.75(1.04)	6.10(0.94)	3.80(1.81)	5.93(1.06)
positive_2_1_3	6.75(0.99)	7.00(0.71)	4.95(1.75)	6.35(0.96)	5.75(1.58)	6.46(0.86)	5.20(0.93)	6.35(0.91)	3.30(1.71)	5.25(1.73)
positive_film_1	6.20(0.87)	6.25(1.04)	4.80(2.05)	5.20(0.98)	5.10(1.10)	4.60(1.39)	4.10(2.54)	5.15(1.24)	1.90(1.02)	4.65(1.56)
positive_film_2	6.10(0.89)	6.00(0.89)	5.60(1.43)	5.60(1.07)	5.95(1.40)	5.95(1.28)	4.85(1.53)	5.50(1.40)	3.60(2.40)	4.25(1.09)
positive_film_3	6.50(1.19)	6.20(0.87)	5.45(1.85)	5.50(1.63)	4.85(2.19)	5.30(1.49)	5.55(1.74)	5.10(1.04)	3.15(3.20)	3.95(1.43)
neutral_1_1_1	5.20(0.40)	5.25(0.94)	2.05(0.74)	2.65(1.15)	6.55(1.12)	4.10(1.22)	6.65(0.85)	5.15(1.19)	3.80(2.56)	3.75(0.94)
neutral_1_1_2	5.05(0.59)	4.85(0.65)	2.90(1.14)	2.95(1.02)	6.00(1.52)	3.90(1.09)	6.30(0.64)	5.40(1.43)	6.05(1.94)	4.60(1.85)
neutral_1_1_3	4.90(0.54)	4.80(0.81)	2.05(1.20)	2.95(0.97)	5.75(1.76)	3.82(1.06)	6.20(1.12)	5.35(0.96)	3.75(2.02)	4.85(1.68)
neutral_2_1_1	5.75(0.83)	5.05(0.50)	2.45(1.40)	2.40(1.46)	5.50(1.57)	6.30(1.14)	4.95(1.07)	6.17(1.13)	3.45(2.11)	5.60(1.85)
neutral_2_1_2	5.15(1.11)	5.15(0.57)	2.55(1.02)	2.20(0.98)	4.75(1.04)	6.00(1.90)	5.25(1.48)	5.70(1.14)	3.60(1.98)	5.95(1.47)
neutral_2_1_3	5.65(0.73)	5.15(0.57)	2.65(1.42)	2.25(0.83)	5.10(1.14)	6.05(1.43)	5.45(0.97)	6.10(0.83)	2.20(1.40)	5.30(1.82)
neutral_film_1	4.70(1.23)	5.40(0.80)	2.70(1.23)	2.45(1.36)	4.65(1.06)	5.15(0.91)	6.00(0.77)	3.67(2.19)	3.70(1.79)	4.20(1.33)
neutral_film_2	4.55(0.97)	4.90(1.09)	2.70(1.49)	2.65(0.96)	5.25(1.04)	5.20(1.40)	5.72(1.99)	5.00(1.61)	3.30(2.00)	4.85(1.77)
neutral_film_3	5.45(0.80)	5.10(0.62)	3.40(1.59)	3.10(1.04)	5.20(1.63)	5.05(1.16)	4.75(1.18)	5.35(1.53)	3.40(1.91)	4.55(1.53)
negative_1_1_1	1.75(0.70)	2.70(0.95)	6.70(1.27)	6.05(1.40)	4.65(2.41)	4.85(1.15)	2.70(1.14)	2.35(1.11)	5.15(1.82)	3.20(1.33)
negative_1_1_2	1.05(0.22)	1.45(0.86)	8.40(0.66)	7.50(0.87)	1.05(0.22)	1.15(0.36)	1.10(0.30)	1.85(0.79)	2.15(2.03)	193(1.08)
negative_1_1_3	2.20(0.98)	1.90(0.89)	7.25(1.13)	6.00(2.10)	2.30(1.55)	3.55(1.96)	2.20(0.93)	2.50(1.02)	2.70(1.45)	2.13(1.17)
negative_2_1_1	2.75(0.93)	1.90(0.77)	5.50(1.20)	7.00(1.14)	3.95(1.32)	5.30(2.12)	2.75(0.83)	2.40(1.07)	4.20(2.52)	4.40(1.62)
negative_2_1_2	1.60(0.86)	1.60(0.73)	6.40(1.36)	6.90(1.04)	1.25(0.43)	1.20(0.40)	1.80(0.87)	1.65(0.79)	3.85(2.39)	2.20(1.03)
negative_2_1_3	2.80(0.98)	2.40(0.73)	6.45(1.16)	6.45(0.97)	3.95(1.77)	5.25(0.99)	2.40(0.86)	2.45(0.80)	4.75(2.93)	5.30(1.23)
negative_film_1	3.60(1.24)	3.30(1.19)	4.65(1.56)	4.00(1.41)	3.85(1.39)	3.95(1.56)	4.70(1.62)	3.14(1.20)	2.65(1.53)	3.60(1.46)
negative_film_2	3.30(1.14)	2.95(0.80)	4.15(1.53)	5.60(0.92)	4.00(1.34)	4.10(1.34)	2.85(1.62)	3.15(1.42)	3.60(2.31)	4.80(1.66)
negative_film_3	2.45(1.32)	3.90(0.99)	4.45(1.66)	4.60(1.46)	3.30(1.35)	4.00(1.48)	3.30(2.19)	3.50(1.24)	1.65(0.73)	3.45(0.92)

## Discussion

This study presents the development of a standardized database of Chinese emotional short videos based on age and gender differences, where both the subjective and physiological responses of participants were measured. In this database, a total of 54 short videos were selected that successfully elicited three categories of emotions for different age and gender groups. We conducted two experiments to validate these short videos as emotion elicitation stimuli and compared our database with the existing Chinese film database. The experimental results give the supports for our three hypotheses: 1) the participants of different ages and genders show different emotional responses when watching emotional short videos; 2) the short videos are reliable audio-visual stimuli and exhibit better emotion elicitation effects compared with film clips; 3) the specific short videos designed for participants with different ages and genders have better emotion elicitation effects than those ignoring age and gender differences. The summary of the database contents is given in Table 6.

**Table 6:** Summary of the database contents.

**Subjective Experience**	
**Number of participants**	360
**Number of short videos**	240
**Short video duration**	71–232 seconds
**Selection method**	manually selected
**Rating scales**	Valence
	Arousal
	Emotion evaluation scale
**Rating values**	Discrete scale of 1–9
**Physiological Response**	
**Number of participants**	81
**Number of short videos**	54
**Short video duration**	91–229 seconds
**Selection method**	Subset of annotated short videos with the highest success index
**Rating scales**	Valence
	Arousal
	Dominance
	Liking
	Familiarity
	Emotion evaluation scale
**Rating values**	Discrete scale of 1–9
**Recorded signals**	channel 256Hz EEG
	GSR
	Face video

We found the age and gender differences do exist when the participants watched emotional short videos, which was consistent with the previous literatures about individual differences in emotional response. As far as the age difference, the participants of three age groups gave different valence scores for short videos of different emotion categories, especially for disgust, fear, tenderness and amusement as shown in Fig 1. While the age difference in arousal was only presented in the emotion category of disgust. This difference by age factor is similar to those reported in several previous studies [27–29]. In fact, the differences in emotional responses brought about by age can be explained from the perspective of cognitive science. Theoretically, the human cognitive system is subject to the changes with age and experience. The conceptual knowledge of emotion at a particular stage is the result of the differentiation of previous conceptual knowledge of emotion by multiple factors. With regard to the gender difference, the female always tends to report stronger emotional responses. This is in line with the results of informed researches that the female reported higher arousal scores and had higher emotional expressivity [30–32]. It is reasonable to speculate that this inconsistency stems from the socialization of humans by gender over a long evolutionary period. The males play the role of going out to hunt and protecting their families, and they must be sensitive to threatening stimuli. The females, on the other hand, are tasked with reproducing offspring, and they need to have a greater ability to recognize the emotions of others and to express their own emotions in order to receive more support and assistance [49]. Although either age difference or gender difference have been confirmed by relevant works, no studies have examined age and gender differences in the same emotional stimuli simultaneously. In our database, the age and gender differences of participants are explored together for the first time. The experiment results show that the age and gender differences appear in both valence and arousal dimensions when watching emotional short videos. This phenomenon can be regarded as a justification for providing different emotional stimuli for different participants.

A major contribution of the current work was to design specific short videos for participants of different ages and genders. The existing emotion elicitation databases have been set up to provide the same stimuli for all participants, however, the participants with different ages and genders extremely likely have different emotional responses to the same stimuli. Therefore, the traditional methods of constructing the emotion elicitation database for all participants cannot guarantee the emotion elicitation effects. Different from the previous studies, our database takes age and gender differences into account and provides specific emotional short videos that can induce three emotions (positive, neutral and negative) for the participants of age (20–24, 25–29 and 30–34) and gender (male and female). In terms of objective physiological signals and subjective evaluation, the experimental results have verified the effectiveness of targeted delivering emotional short videos based on individual differences.

Another contribution of this work was the concurrent measurements of subjective and physiological responses. Most of the emotion elicitation databases were established by means of recording and analyzing the subjective evaluation results. Inevitably, limited by individual subjective consciousness, the subjective methods are difficult to objectively reflect the real emotional state of participants. So far as we know, only one Chinese emotion elicitation database had recorded the physiological responses of heart rate and respiration rate [24]. Compared with other physiological signals, the EEG signals directly record the changes in scalp potential, which can reflect the real emotional state of participants more reliably. Pursuing the realistic emotional states of participants, we used the Emotiv EPOC X equipment and EmotivPRO software to capture and record the EEG signals of participants while watching video stimuli. The collected EEG signals can be used to objectively evaluate the eliciting effects of emotional stimulus database in conjunction with subjective evaluation.

As mentioned above, the current study extends the previous literatures on emotion elicitation database in several ways. However, there are still some limitations worth noting. Firstly in our study, more than half of the participants were aged 20–24, while the numbers of participants aged 25–29 and 30–34 were relatively small. In order to improve the generalizability of the findings, we would expand the sample size of participants aged 25–34 in future work. Secondly, our work was carried out on the participants among the age range of 20–34, the future studies should expand the age range of the database and make it applicable to the wider range of age groups. Thirdly during the experiments, we recorded the EEG signals, GSR and face videos as physiological signals, but only the EEG signals were used to perform the emotion recognition. In the future, we can further analyze multimodal physiological signals such as GSR and face videos, so as to obtain more comprehensive and accurate emotion analysis results. Fourthly, eliciting emotions in a participant depends on a number of factors, such as age, gender, experience of the person, familiarity with the video and so on. We have done a pilot study to explore the age and gender differences in emotional responses when watching short videos, the effects of other factors, such as experience of the person and familiarity with the video, will be further considered in the future work. Last but not the least, our database was built for the Chinese participants, which may induce different types of emotions or emotional intensity for participants from other nationalities. But that does not mean our database cannot be used to elicit emotions for other nationalities, it also allows researchers to compare the emotional responses of participants from different nationalities.

## Conclusion

This paper develops a standardized database of Chinese emotional short videos based on age and gender differences. By analyzing the valence and arousal scores of participants in the subjective evaluations, we found that there were indeed age and gender differences when the participants watched emotional short videos. Inspired by this, the specific short videos were also designed for participants of different ages and genders in order to achieve better emotional elicitation effects. As a result, a total of 54 emotional short videos were selected for 6 groups of participants, including the male and female respectively aged in 20–24, 25–29 and 30–34. In particular, the participants in each group were matched with 9 specific short videos, including 3 positive, 3 neutral and 3 negative stimuli. Both the subjective experiences and physiological responses were recorded and analyzed to validate the effectiveness of our emotional short video database.

## Supporting information

S1 Video The supporting information of this paper can be downloaded at the following link, https://github.com/EEG-Emotion-Recognition-group/A-standardized-database-of-Chineseemotional-short-videos-based-on-age-and-gender-differences.git. (ZIP)

## References

[1] Grundmann F, Epstude K, Scheibe S. Face masks reduce emotion-recognition accuracy and perceived closeness. Plos One. 2021;16(4):e0249792. doi: 10.1371/journal.pone.0249792 33891614 PMC8064590

[2] Kobai R, Murakami H. Effects of interactions between facial expressions and self-focused attention on emotion. Plos One. 2021;16(12):e0261666. doi: 10.1371/journal.pone.0261666 34941917 PMC8699986

[3] Mastria S, Agnoli S, Corazza GE. How does emotion influence the creativity evaluation of exogenous alternative ideas? PloS One. 2019;14(7):e0219298. doi: 10.1371/journal.pone.0219298 31276480 PMC6611619

[4] Puviani L, Rama S, Vitetta GM. A mathematical description of emotional processes and its potential applications to affective computing. IEEE Transactions on Affective Computing. 2021;12(3):692–706. doi: 10.1109/TAFFC.2018.2887385

[5] Gross JJ, Levenson RW. Emotion elicitation using films. Cognition & emotion. 1995;9(1):87–108. doi: 10.1080/02699939508408966

[6] Lang PJ, Bradley MM, Cuthbert BN, et al. International affective picture system (IAPS): technical manual and affective ratings. NIMH Center for the Study of Emotion and Attention. 1997;1(3):39–58.

[7] Redondo J, Fraga I, Padrón I, Comesaña M. The Spanish adaptation of ANEW (affective norms for English words). Behavior Research Methods. 2007;39(3):600–605. doi: 10.3758/BF03193031 17958173

[8] Fernández C, Pascual JC, Soler J, Elices M, Portella MJ, Fernández-Abascal E. Physiological responses induced by emotion-eliciting films. Applied Psychophysiology and Biofeedback. 2012;37(2):73–79. doi: 10.1007/s10484-012-9180-7 22311202

[9] Kring AM, Gordon AH. Sex differences in emotion: expression, experience, and physiology. Journal of Personality and Social Psychology. 1998;74(3):686. doi: 10.1037/0022-3514.74.3.686 9523412

[10] Gerrards-Hesse A, Spies K, Hesse FW. Experimental inductions of emotional states and their effectiveness: a review. British Journal of Psychology. 1994;85(1):55–78. doi: 10.1111/j.2044-8295.1994.tb02508.x

[11] Harmon-Jones E, Harmon-Jones C, Summerell E. On the importance of both dimensional and discrete models of emotion. Behavioral Sciences. 2017;7(4):66. doi: 10.3390/bs7040066 28961185 PMC5746675

[12] Lang PJ, Greenwald MK, Bradley MM, Hamm AO. Looking at pictures: affective, facial, visceral, and behavioral reactions. Psychophysiology. 1993;30(3):261–273. doi: 10.1111/j.1469-8986.1993.tb03352.x 8497555

[13] Baveye Y, Dellandrea E, Chamaret C, Chen L. LIRIS-ACCEDE: a video database for affective content analysis. IEEE Transactions on Affective Computing. 2015;6(1):43–55. doi: 10.1109/TAFFC.2015.2396531

[14] Zheng WL, Lu BL. Investigating critical frequency bands and channels for EEG-based emotion recognition with deep neural networks. IEEE Transactions on Autonomous Mental Development. 2015;7(3):162–175. doi: 10.1109/TAMD.2015.2431497

[15] Ismail S, Aziz NAA, Ibrahim SZ, Khan CT, Rahman MA. Selecting video stimuli for emotion elicitation via online survey. Human-Centric Computing and Information Sciences. 2021;11(36):1–18.

[16] Philippot P. Inducing and assessing differentiated emotion-feeling states in the laboratory. Cognition and Emotion. 1993;7(2):171–193. doi: 10.1080/02699939308409183 27102736

[17] Ekman P, Friesen WV, O’sullivan M, Chan A, Diacoyanni-Tarlatzis I, Heider K, et al. Universals and cultural differences in the judgments of facial expressions of emotion. Journal of Personality and Social Psychology. 1987;53(4):712. doi: 10.1037/0022-3514.53.4.712 3681648

[18] Jeong D, Han SH, Jeong DY, Kwon K. Building a database of 4D movie clips eliciting affect/emotions. In: Joint Conference of the Asian Council on Ergonomics and Design and the Southeast Asian Network of Ergonomics Societies. Springer; 2020. pp. 3–7.

[19] Keltner D, Tracy JL, Sauter D, Cowen A. What basic emotion theory really says for the twenty-first century study of emotion. Journal of Nonverbal Behavior. 2019;43(2):195–201. doi: 10.1007/s10919-019-00298-y 31404243 PMC6688640

[20] Senft N, Campos B, Shiota MN, Chentsova-Dutton YE. Who emphasizes positivity? An exploration of emotion values in people of Latino, Asian, and European heritage living in the United States. Emotion. 2021;21(4):707. doi: 10.1037/emo0000737 32191097

[21] Tsai W, Lu Q. Culture, emotion suppression and disclosure, and health. Social and Personality Psychology Compass. 2018;12(3):e12373. doi: 10.1111/spc3.12373

[22] Michelini Y, Acuña I, Guzmán JI, Godoy JC. LATEMO-E: a film database to elicit discrete emotions and evaluate emotional dimensions in Latin-Americans. Trends in Psychology. 2019;27:473–490.

[23] Shalchizadeh F, Shamekhi S, Sadeh RN, Darvish A. Persian emotion elicitation film set and signal database. Biomedical Signal Processing and Control. 2022;72:103290. doi: 10.1016/j.bspc.2021.103290

[24] Deng Y, Yang M, Zhou R. A new standardized emotional film database for Asian culture. Frontiers in Psychology. 2017;8:1941. doi: 10.3389/fpsyg.2017.01941 29163312 PMC5675887

[25] Xu P, Huang Y, Luo Y. Establishment and assessment of native Chinese affective video system. Chinese Mental Health Journal. 2010;24(7):551–554.

[26] Zhang Y, Zhao G, Shu Y, Ge Y, Zhang D, Liu YJ, et al. CPED: a Chinese positive emotion database for emotion elicitation and analysis. IEEE Transactions on Affective Computing. 2021. doi: 10.1007/978-3-030-78961-9

[27] Mather M, Canli T, English T, Whitfield S, Wais P, Ochsner K, et al. Amygdala responses to emotionally valenced stimuli in older and younger adults. Psychological Science. 2004;15(4):259–263. doi: 10.1111/j.0956-7976.2004.00662.x 15043644

[28] Burriss L, Powell D, White J. Psychophysiological and subjective indices of emotion as a function of age and gender. Cognition and Emotion. 2007;21(1):182–210. doi: 10.1080/02699930600562235

[29] Jenkins LM, Andrewes DG. A new set of standardised verbal and non-verbal contemporary film stimuli for the elicitation of emotions. Brain Impairment. 2012;13(2):212–227. doi: 10.1017/BrImp.2012.18

[30] Bradley MM, Codispoti M, Sabatinelli D, Lang PJ. Emotion and motivation II: sex differences in picture processing. Emotion. 2001;1(3):300. doi: 10.1037/1528-3542.1.3.300 12934688

[31] Gohier B, Senior C, Brittain P, Lounes N, El-Hage W, Law V, et al. Gender differences in the sensitivity to negative stimuli: cross-modal affective priming study. European Psychiatry. 2013;28(2):74–80. doi: 10.1016/j.eurpsy.2011.06.007 21908178

[32] Deng Y, Chang L, Yang M, Huo M, Zhou R. Gender differences in emotional response: Inconsistency between experience and expressivity. PloS One. 2016;11(6):e0158666. doi: 10.1371/journal.pone.0158666 27362361 PMC4928818

[33] Dzedzickis A, Kaklauskas A, Bucinskas V. Human emotion recognition: review of sensors and methods. Sensors. 2020;20(3):592. doi: 10.3390/s20030592 31973140 PMC7037130

[34] Suhaimi NS, Mountstephens J, Teo J. EEG-based emotion recognition: a state-of-the-art review of current trends and opportunities. Computational Intelligence and Neuroscience. 2020. doi: 10.1155/2020/8875426 33014031 PMC7516734

[35] Song T, Zheng W, Song P, Cui Z. EEG emotion recognition using dynamical graph convolutional neural networks. IEEE Transactions on Affective Computing. 2018;11(3):532–541. doi: 10.1109/TAFFC.2018.2817622

[36] Montoya-Martínez J, Vanthornhout J, Bertrand A, Francart T. Effect of number and placement of EEG electrodes on measurement of neural tracking of speech. Plos One. 2021;16(2):e0246769. doi: 10.1371/journal.pone.0246769 33571299 PMC7877609

[37] Koelstra S, Muhl C, Soleymani M, Lee JS, Yazdani A, Ebrahimi T, et al. Deap: a database for emotion analysis; using physiological signals. IEEE Transactions on Affective Computing. 2011;3(1):18–31. doi: 10.1109/T-AFFC.2011.15

[38] Xu X, Jia T, Li Q, Wei F, Ye L, Wu X. EEG feature selection via global redundancy minimization for emotion recognition. IEEE Transactions on Affective Computing. 2021.

[39] Dadebayev D, Goh WW, Tan EX. EEG-based emotion recognition: review of commercial EEG devices and machine learning techniques. Journal of King Saud University-Computer and Information Sciences. 2021.

[40] Liu YJ, Yu M, Zhao G, Song J, Ge Y, Shi Y. Real-time movie-induced discrete emotion recognition from EEG signals. IEEE Transactions on Affective Computing. 2017;9(4):550–562. doi: 10.1109/TAFFC.2017.2660485

[41] Chatzichronis S, Alexiou A, Simou P, Mantzavinos V, Tsiamis V, et al. Neurocognitive assessment software for enrichment sensory environments. J Proteomics Bioinform. 2019;12:18–28.

[42] Damen D, Doughty H, Farinella GM, Fidler S, Furnari A, Kazakos E, et al. Scaling egocentric vision: The epic-kitchens dataset. In: Proceedings of the European Conference on Computer Vision; 2018. pp. 720–736.

[43] Caba Heilbron F, Escorcia V, Ghanem B, Carlos Niebles J. Activitynet: A large-scale video benchmark for human activity understanding. In: Proceedings of the IEEE Conference on Computer Vision and Pattern Recognition; 2015. pp. 961–970.

[44] Liu Y, Peng B, Shi P, Yan H, Zhou Y, Han B, et al. iqiyi-vid: A large dataset for multi-modal person identification. arXiv:181107548. 2018.

[45] Ge Y, Zhao G, Zhang Y, Houston RJ, Song J. A standardised database of Chinese emotional film clips. Cognition and Emotion. 2019;33(5):976–990. doi: 10.1080/02699931.2018.1530197 30293475

[46] Qian M, Wu G, Zhu R, Zhang S. Development of the revised Eysenck personality questionnaire short scale for Chinese (EPQ-RSC). Acta Psychologica Sinica. 2000;32(3):317.

[47] Bradley MM, Lang PJ. Measuring emotion: the self-assessment manikin and the semantic differential. Journal of Behavior Therapy and Experimental Psychiatry. 1994;25(1):49–59. doi: 10.1016/0005-7916(94)90063-9 7962581

[48] Duan RN, Zhu JY, Lu BL. Differential entropy feature for EEG-based emotion classification. In: 2013 6th International IEEE/EMBS Conference on Neural Engineering. 2013. pp. 81–84.

[49] Fischer AH, Kret ME, Broekens J. Gender differences in emotion perception and self-reported emotional intelligence: a test of the emotion sensitivity hypothesis. PloS One. 2018;13(1):e0190712. doi: 10.1371/journal.pone.0190712 29370198 PMC5784910

