# Supplementary material for: A standardized database of Chinese emotional short videos based on age and gender differences
Source: PLoS One. 2023 Mar 30;18(3):e0283573. doi: 10.1371/journal.pone.0283573 (PMC10062648; doi:10.1371/journal.pone.0283573)
Supplement: S1 Video — (ZIP) [file pone.0283573.s001.zip › supporting information/All the data.zip!/All the data/Experiment 2/Statistical analysis results on SAM scale of male and female groups aged 20-24.docx]

**Table 6. The statistical analysis results on SAM scale of male and female groups aged 20-24.**

|  | VAL | | ARO | | LIK | | DOM | | FAM | |
| --- | --- | --- | --- | --- | --- | --- | --- | --- | --- | --- |
| Stimulus Name | Male | Female | Male | Female | Male | Female | Male | Female | Male | Female |
| positive_1_1_1 | 7.45(1.02) | 6.50(0.97) | 6.95(0.92) | 6.20(1.50) | 7.20(1.12) | 5.53(1.02) | 6.80(0.87) | 5.95(0.92) | 6.20(1.72) | 3.85(1.56) |
| positive_1_1_2 | 7.50(1.16) | 6.40(1.11) | 6.70(0.95) | 6.10(1.30) | 6.65(1.39) | 4.85(1.96) | 6.45(1.13) | 5.10(1.76) | 5.00(2.47) | 2.12(1.31) |
| positive_1_1_3 | 7.50(0.92) | 6.80(0.87) | 6.85(0.96) | 6.00(0.63) | 7.20(1.25) | 5.95(1.43) | 6.35(1.46) | 5.65(1.28) | 5.60(2.62) | 4.85(1.49) |
| positive_2_1_1 | 6.65(0.85) | 7.35(0.85) | 6.30(1.05) | 7.15(0.91) | 6.40(1.24) | 7.80(0.62) | 6.15(1.01) | 6.45(1.07) | 3.55(1.86) | 6.70(1.00) |
| positive_2_1_2 | 7.45(0.67) | 7.08(0.83) | 6.85(1.28) | 6.80(0.81) | 5.55(1.02) | 6.85(1.06) | 5.75(1.04) | 6.10(0.94) | 3.80(1.81) | 5.93(1.06) |
| positive_2_1_3 | 6.75(0.99) | 7.00(0.71) | 4.95(1.75) | 6.35(0.96) | 5.75(1.58) | 6.46(0.86) | 5.20(0.93) | 6.35(0.91) | 3.30(1.71) | 5.25(1.73) |
| positive_film_1 | 6.20(0.87) | 6.25(1.04) | 4.80(2.05) | 5.20(0.98) | 5.10(1.10) | 4.60(1.39) | 4.10(2.54) | 5.15(1.24) | 1.90(1.02) | 4.65(1.56) |
| positive_film_2 | 6.10(0.89) | 6.00(0.89) | 5.60(1.43) | 5.60(1.07) | 5.95(1.40) | 5.95(1.28) | 4.85(1.53) | 5.50(1.40) | 3.60(2.40) | 4.25(1.09) |
| positive_film_3 | 6.50(1.19) | 6.20(0.87) | 5.45(1.85) | 5.50(1.63) | 4.85(2.19) | 5.30(1.49) | 5.55(1.74) | 5.10(1.04) | 3.15(3.20) | 3.95(1.43) |
| neutral_1_1_1 | 5.20(0.40) | 5.25(0.94) | 2.05(0.74) | 2.65(1.15) | 6.55(1.12) | 4.10(1.22) | 6.65(0.85) | 5.15(1.19) | 3.80(2.56) | 3.75(0.94) |
| neutral_1_1_2 | 5.05(0.59) | 4.85(0.65) | 2.90(1.14) | 2.95(1.02) | 6.00(1.52) | 3.90(1.09) | 6.30(0.64) | 5.40(1.43) | 6.05(1.94) | 4.60(1.85) |
| neutral_1_1_3 | 4.90(0.54) | 4.80(0.81) | 2.05(1.20) | 2.95(0.97) | 5.75(1.76) | 3.82(1.06) | 6.20(1.12) | 5.35(0.96) | 3.75(2.02) | 4.85(1.68) |
| neutral_2_1_1 | 5.75(0.83) | 5.05(0.50) | 2.45(1.40) | 2.40(1.46) | 5.50(1.57) | 6.30(1.14) | 4.95(1.07) | 6.17(1.13) | 3.45(2.11) | 5.60(1.85) |
| neutral_2_1_2 | 5.15(1.11) | 5.15(0.57) | 2.55(1.02) | 2.20(0.98) | 4.75(1.04) | 6.00(1.90) | 5.25(1.48) | 5.70(1.14) | 3.60(1.98) | 5.95(1.47) |
| neutral_2_1_3 | 5.65(0.73) | 5.15(0.57) | 2.65(1.42) | 2.25(0.83) | 5.10(1.14) | 6.05(1.43) | 5.45(0.97) | 6.10(0.83) | 2.20(1.40) | 5.30(1.82) |
| neutral_film_1 | 4.70(1.23) | 5.40(0.80) | 2.70(1.23) | 2.45(1.36) | 4.65(1.06) | 5.15(0.91) | 6.00(0.77) | 3.67(2.19) | 3.70(1.79) | 4.20(1.33) |
| neutral_film_2 | 4.55(0.97) | 4.90(1.09) | 2.70(1.49) | 2.65(0.96) | 5.25(1.04) | 5.20(1.40) | 5.72(1.99) | 5.00(1.61) | 3.30(2.00) | 4.85(1.77) |
| neutral_film_3 | 5.45(0.80) | 5.10(0.62) | 3.40(1.59) | 3.10(1.04) | 5.20(1.63) | 5.05(1.16) | 4.75(1.18) | 5.35(1.53) | 3.40(1.91) | 4.55(1.53) |
| negative_1_1_1 | 1.75(0.70) | 2.70(0.95) | 6.70(1.27) | 6.05(1.40) | 4.65(2.41) | 4.85(1.15) | 2.70(1.14) | 2.35(1.11) | 5.15(1.82) | 3.20(1.33) |
| negative_1_1_2 | 1.05(0.22) | 1.45(0.86) | 8.40(0.66) | 7.50(0.87) | 1.05(0.22) | 1.15(0.36) | 1.10(0.30) | 1.85(0.79) | 2.15(2.03) | 193(1.08) |
| negative_1_1_3 | 2.20(0.98) | 1.90(0.89) | 7.25(1.13) | 6.00(2.10) | 2.30(1.55) | 3.55(1.96) | 2.20(0.93) | 2.50(1.02) | 2.70(1.45) | 2.13(1.17) |
| negative_2_1_1 | 2.75(0.93) | 1.90(0.77) | 5.50(1.20) | 7.00(1.14) | 3.95(1.32) | 5.30(2.12) | 2.75(0.83) | 2.40(1.07) | 4.20(2.52) | 4.40(1.62) |
| negative_2_1_2 | 1.60(0.86) | 1.60(0.73) | 6.40(1.36) | 6.90(1.04) | 1.25(0.43) | 1.20(0.40) | 1.80(0.87) | 1.65(0.79) | 3.85(2.39) | 2.20(1.03) |
| negative_2_1_3 | 2.80(0.98) | 2.40(0.73) | 6.45(1.16) | 6.45(0.97) | 3.95(1.77) | 5.25(0.99) | 2.40(0.86) | 2.45(0.80) | 4.75(2.93) | 5.30(1.23) |
| negative_film_1 | 3.60(1.24) | 3.30(1.19) | 4.65(1.56) | 4.00(1.41) | 3.85(1.39) | 3.95(1.56) | 4.70(1.62) | 3.14(1.20) | 2.65(1.53) | 3.60(1.46) |
| negative_film_2 | 3.30(1.14) | 2.95(0.80) | 4.15(1.53) | 5.60(0.92) | 4.00(1.34) | 4.10(1.34) | 2.85(1.62) | 3.15(1.42) | 3.60(2.31) | 4.80(1.66) |
| negative_film_3 | 2.45(1.32) | 3.90(0.99) | 4.45(1.66) | 4.60(1.46) | 3.30(1.35) | 4.00(1.48) | 3.30(2.19) | 3.50(1.24) | 1.65(0.73) | 3.45(0.92) |
